# Decrease of α-defensin impairs intestinal metabolite homeostasis via dysbiosis in mouse chronic social defeat stress model

**DOI:** 10.1038/s41598-021-89308-y

**Published:** 2021-05-10

**Authors:** Kosuke Suzuki, Kiminori Nakamura, Yu Shimizu, Yuki Yokoi, Shuya Ohira, Mizu Hagiwara, Yi Wang, Yuchi Song, Tomoyasu Aizawa, Tokiyoshi Ayabe

**Affiliations:** 1 Innate Immunity Laboratory, Graduate School of Life Science, Hokkaido University, Sapporo, Japan; 2 Department of Cell Biological Science, Faculty of Advanced Life Science, Hokkaido University, Sapporo, 001-0021 Japan; 3 Laboratory of Protein Science, Department of Advanced Transdisciplinary Science, Faculty of Advanced Life Science, Hokkaido University, Sapporo, Japan; 4 Global Station for Soft Matter, Global Institution for Collaborative Research and Education, Hokkaido University, Sapporo, Japan

**Keywords:** Microbiome, Mucosal immunology, Neurological disorders, Experimental models of disease

## Abstract

Psychological stress has been reported to relate to dysbiosis, imbalance of the intestinal microbiota composition, and contribute to the onset and exacerbation of depression, though, underlying mechanisms of psychological stress-related dysbiosis have been unknown. It has been previously established that α-defensins, which are effector peptides of innate enteric immunity produced by Paneth cells in the small intestine, play an important role in regulation of the intestinal microbiota. However, the relationship between disruption of intestinal ecosystem and α-defensin under psychological stress is yet to be determined. Here we show using chronic social defeat stress (CSDS), a mouse depression model that (1) the exposure to CSDS significantly reduces α-defensin secretion by Paneth cells and (2) induces dysbiosis and significant composition changes in the intestinal metabolites. Furthermore, (3) they are recovered by administration of α-defensin. These results indicate that α-defensin plays an important role in maintaining homeostasis of the intestinal ecosystem under psychological stress, providing novel insights into the onset mechanism of stress-induced depression, and may further contribute to discovery of treatment targets for depression.

## Introduction

Depression is a serious illness that tends to persist and often relapse, and the number of the patients has been increasing worldwide to cause great social loss^1^. It has been known that multiple factors are involved in the onset of depression such as the decreased monoamine neurotransmitters including dopamine, the overactivation of hypothalamic–pituitary–adrenal (HPA) axis, and decreased neuroplasticity due to combination of genetic factors and psychological stress^2^. Among them, psychological stress is considered an important factor contributing to the development of depression^3^. However, since mechanisms of depression are diverse, the whole picture involving its onset has been still unclear. Relationships between the intestinal microbiota and depression have been reported, recently. The intestinal microbiota has been known to affect immunity of the host via their cell components such as lipopolysaccharide (LPS) or by producing metabolites such as short-chain fatty acids and neurotransmitters including γ-aminobutyric acid (GABA) and serotonin, resulting in changes of brain function to develop depression^4^. In addition, a cohort study showed that a positive correlation between quality of life of patients with depression and the butyrate-producing bacteria or the synthetic potential of bacterial dopamine metabolites^5^. Furthermore, it has been reported that transplanting feces of depression patients into mice produces abnormalities in bacterial tryptophan metabolism and production of short-chain fatty acids and further induces behavior characteristic of depression^6^. Germ-free mice exposed to psychological stress showed the excessive response via HPA axis^7^. It is known that dysbiosis and changes of the microbial metabolites leading to depression are induced by psychological stress^8^. However, underlying mechanisms that psychological stress causes the disruption of homeostasis in the bacterial metabolites due to dysbiosis remains to be determined.

α-Defensin, an antimicrobial peptide, produced and secreted by Paneth cells in the crypt of the small intestine is responsible, in part, for innate enteric immunity^9–12^ and plays a critical role in both elimination and symbiosis in the intestine by killing pathogens while eliciting less bactericidal activities against commensal bacteria^13^. It has been reported that the absence of active α-defensin alters the composition of small intestinal microbiota in mice^14^, and oral administration of α-defensin rescues severe dysbiosis of graft-versus-host disease (GVHD) model mice^15^, indicating that α-defensin plays an important role in maintaining homeostasis of the intestinal microbiota. In addition, it has been known that α-defensin abnormalities cause dysbiosis and disruption of the intestinal metabolism in Crohn’s disease model mice^16, 17^. Dysbiosis caused by Paneth cell damage with decreased α-defensin has been shown to relate to various diseases^18, 19^. Thus, it is possible that α-defensin secreted by Paneth cells contributes to the maintenance of systemic homeostasis by regulating the intestinal microbiota and their metabolites. However, relationships between Paneth cell α-defensin and dysbiosis or disruption of homeostasis in the intestinal metabolites in depression have been unknown.

Here, we provide evidence that a decrease of α-defensin due to psychological stress induces dysbiosis and subsequent disruption of homeostasis in microbial metabolites in CSDS model mice, a model of psychological stress-induced depression, and that α-defensin rescue attenuates the observed imbalance of the intestinal microbiota and their metabolites. This study provides new insights into the mechanism of depression and further contributes to the prevention and the discovery of therapeutic targets for depression.

## Results

### CSDS exposure decreases α-defensin secretion

**Figure 1:**
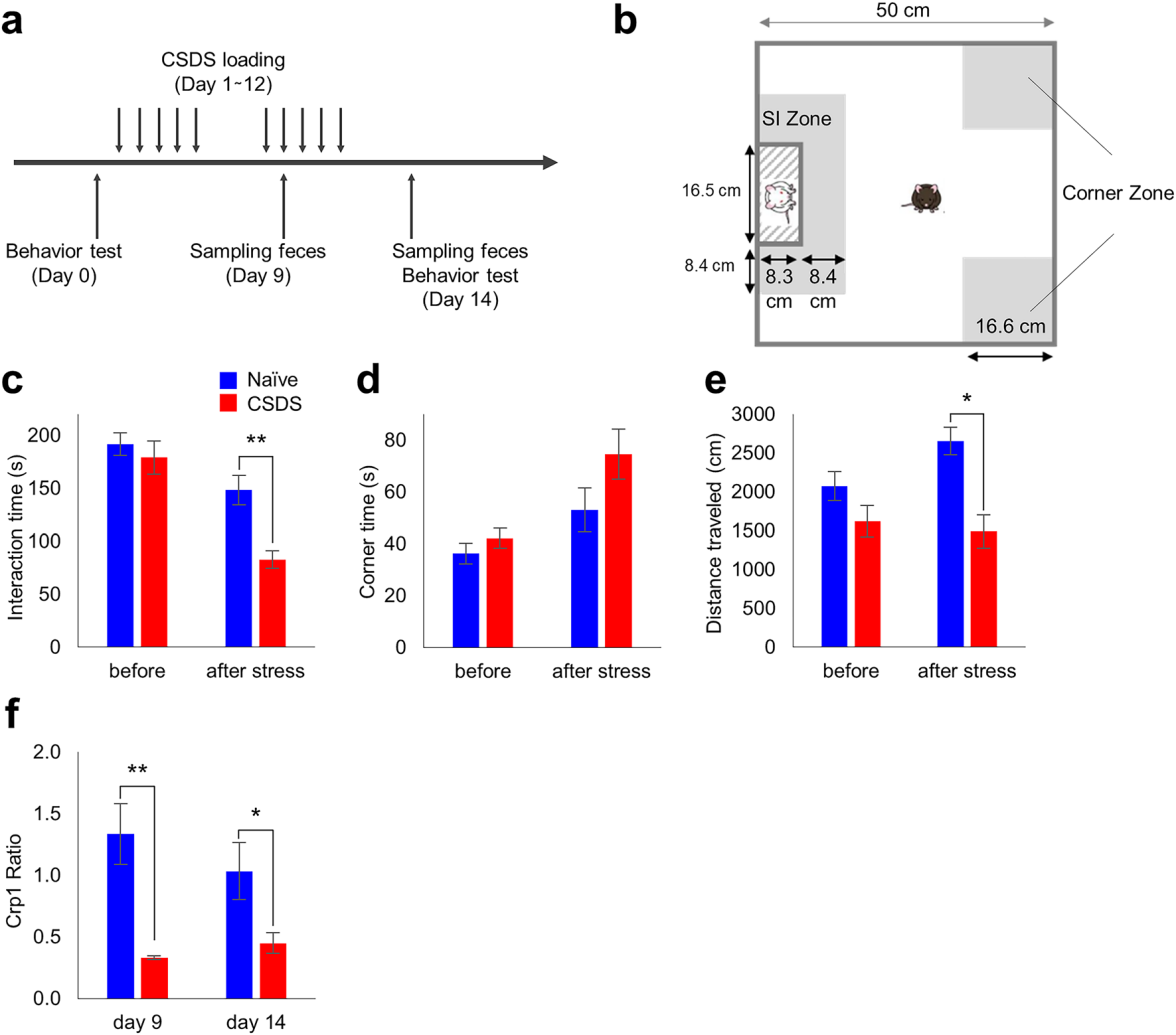
Behavior and fecal cryptdin-1 in CSDS model. (**a**) Schedule of experiment. (**b**) The cage for behavior test. (**c**) Staying time in SI zone. (**d**) Staying time in corner zone. (**e**) Total distance traveled. (**f**) Fecal Crp1 protein ratio vs naïve group in day 1. The data of (**c**)–(**f**) were shown as mean ± SE of six independent experiments in each group. Mann–Whitney U tests were used to compare the data. *P < 0.05; **P < 0.01.

Analysis of behavior changes in the CSDS model (Fig. 1a,b) showed that the interaction time is significantly reduced in CSDS group (82.8 ± 20.3 s.) compared to naïve group (148.5 ± 34.2 s.) (Fig. 1c). In addition, the corner time increased from 53.1 ± 20.7 s. to 74.6 ± 23.8 s. (*P* = 0.093) (Fig. 1d), and the total action distance significantly decreased from 2654.8 ± 439.6 cm to 1490.7 ± 519.3 cm (Fig. 1e). These results indicated that it was confirmed that a characteristic decrease in sociality occurs in the CSDS model.

Next, to analyze the relationship between the CSDS load and the amount of α-defensin secretion, Crp1 in feces of the CSDS (for 12 days) group was measured. The Crp1 amounts on day 9 and day 14 of the CSDS group were decreased to 33% and 45%, respectively, compared to those on day 1 of the naïve group (Fig. 1f, Table S1).

**Figure 2:**
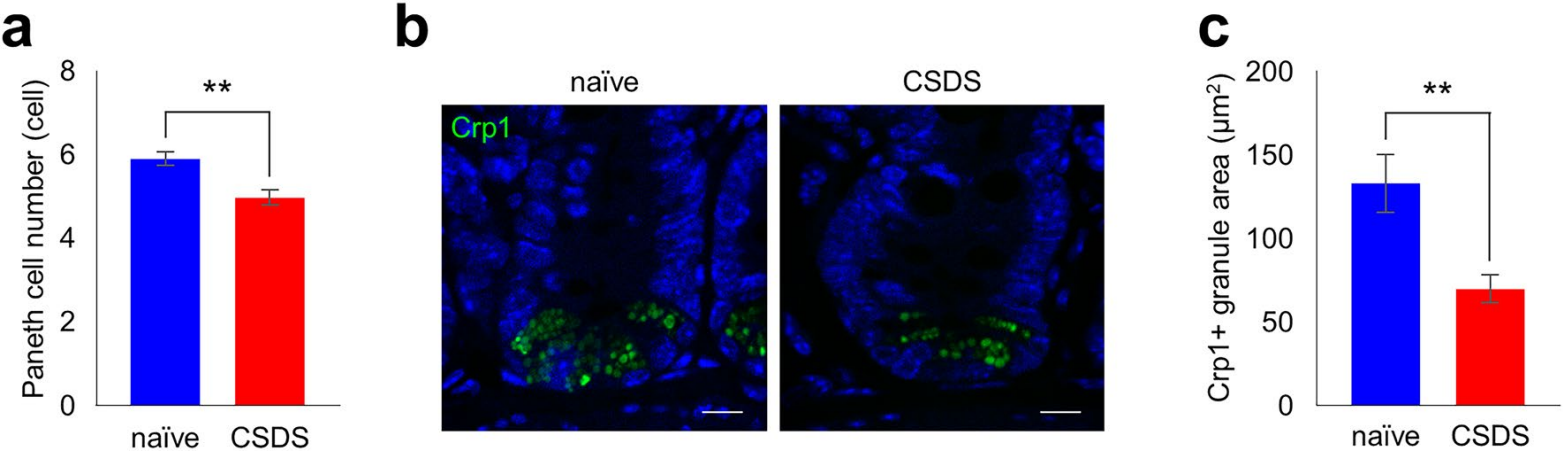
Decrease of Paneth cell number and cryptdin-1 expression in CSDS model. (**a**) Paneth cell number per crypt. (**b**) Representative confocal images of Crp1 (green) with DAPI (blue) of crypt sections. Scale bars indicate 10 µm. (**c**) Crp1 positive granule area per crypt. The data of a, c were shown as mean ± SE of six independent experiments in each group. Mann–Whitney U tests were used to compare the data. **P < 0.01.

We further conducted immunofluorescent analyses of the small intestinal tissue and found a significant decrease in the number of Paneth cells and Crp1^+^ granule area in CSDS group (Fig. 2a–c) compared to naïve group. These results were consistent with the decrease in fecal Crp1 in CSDS group (Fig. 1f).

### Dysbiosis is induced by α-defensin reduction in CSDS model and recovered by Crp4 administration

**Figure 3:**
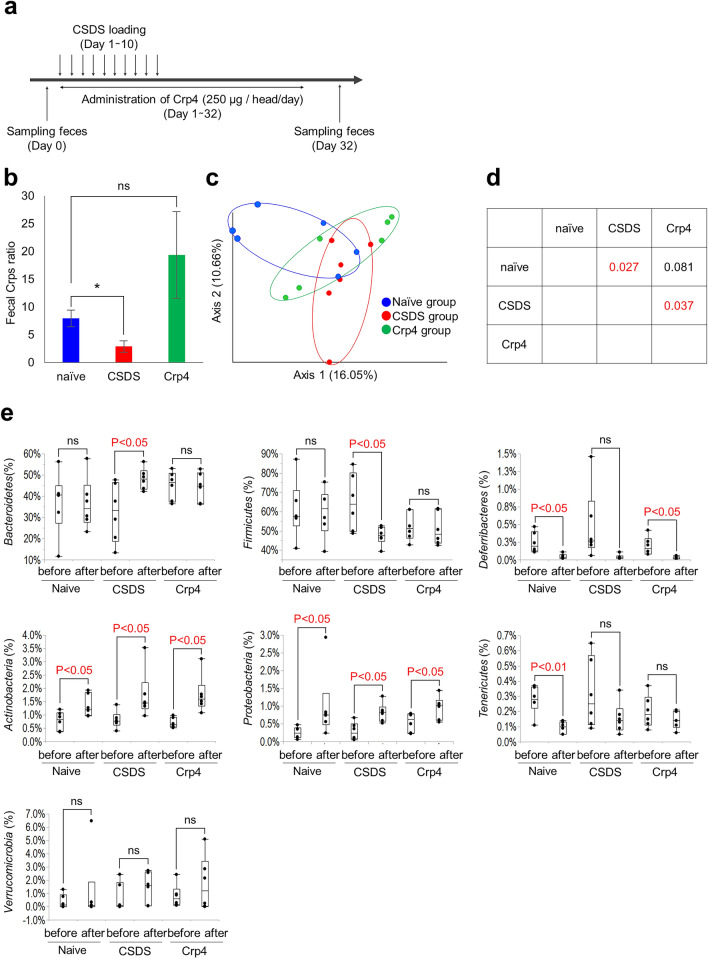
Change in the composition of gut microbiota in the CSDS model by α-defensin administration. (**a**) Schedule of experiment. (**b**) Fecal Crp1 + Crp4 (Crps) protein ratio in day 32 vs before CSDS. Data are shown as the mean ± SE. Steel’s test was used to compare the data (vs naïve group). *P < 0.05. (**c**) PCoA of β-diversity comparison. (**d**) The *P*-values of PERMANOVA test. (**e**) Changes in the genus compositions before or after CSDS (shown only > 0.1% abundance). Pairs student’s t test was used to compare the data. *P < 0.05. The data of b, c, e were shown of six independent experiments in each group.

Next, in order to clarify whether the intestinal microbiota changes due to CSDS and the changes depend on decreased α-defensin, Crp4 administration experiment to rescue α-defensin was conducted focusing on the initial phase under the short stress period (Fig. 3a). The three groups; naïve group, CSDS-loaded group (CSDS group), and CSDS-loaded plus Crp4 administration group (Crp4 group) were analyzed. In the CSDS group, fecal α-defensin was significantly decreased compared to the naïve group (Fig. 3b), consistent with data in our previous experiment (Fig. 1f). In contrast, no significant difference was observed between naïve group and Crp4 group, indicating α-defensin (Crps; Crp1 and Crp4) was rescued by oral administration of Crp4 (Fig. 3b). We analyzed the intestinal microbiota of CSDS group and Crp4 group, and β-diversity showed significant differences between naïve group and CSDS group (*P* = 0.027) and between CSDS group and Crp4 group (*P* = 0.037). In contrast, no difference was observed between naïve group and Crp4 group (Fig. 3c,d), suggesting that dysbiosis caused by CSDS may be recovered by Crp4 administration. We next analyzed the intestinal microbiota composition at Phylum level in the three groups before and after CSDS (Fig. 3e). *Bacteroidetes* increased significantly (from 32 to 48%, *P* = 0.04) and *Firmicutes* decreased significantly (from 65 to 48%, *P* = 0.03) in CSDS group. *Deferribacteres* did not change in CSDS group before and after CSDS loading, whereas significantly decreased in the naïve group (from 0.25 to 0.05%, *P* = 0.02) and Crp4 group (from 0.19 to 0.03%, *P* = 0.03). These data indicated that the characteristic changes of *Bacteroidetes, Firmicutes*, and *Deferribacteres* by CSDS loading were canceled by Crp4 administration. *Actinobacteria* and *Proteobacteria* increased significantly in all groups after CSDS loading. *Tenericutes* decreased significantly only in naïve group (from 0.3 to 0.1%, *P* = 0.001). *Verrucomicrobia* did not change before and after CSDS loading in all groups. These results indicated that CSDS-related dysbiosis is partially rescued by the Cpr4 administration toward the intestinal microbiota in naïve group.

**Figure 4:**
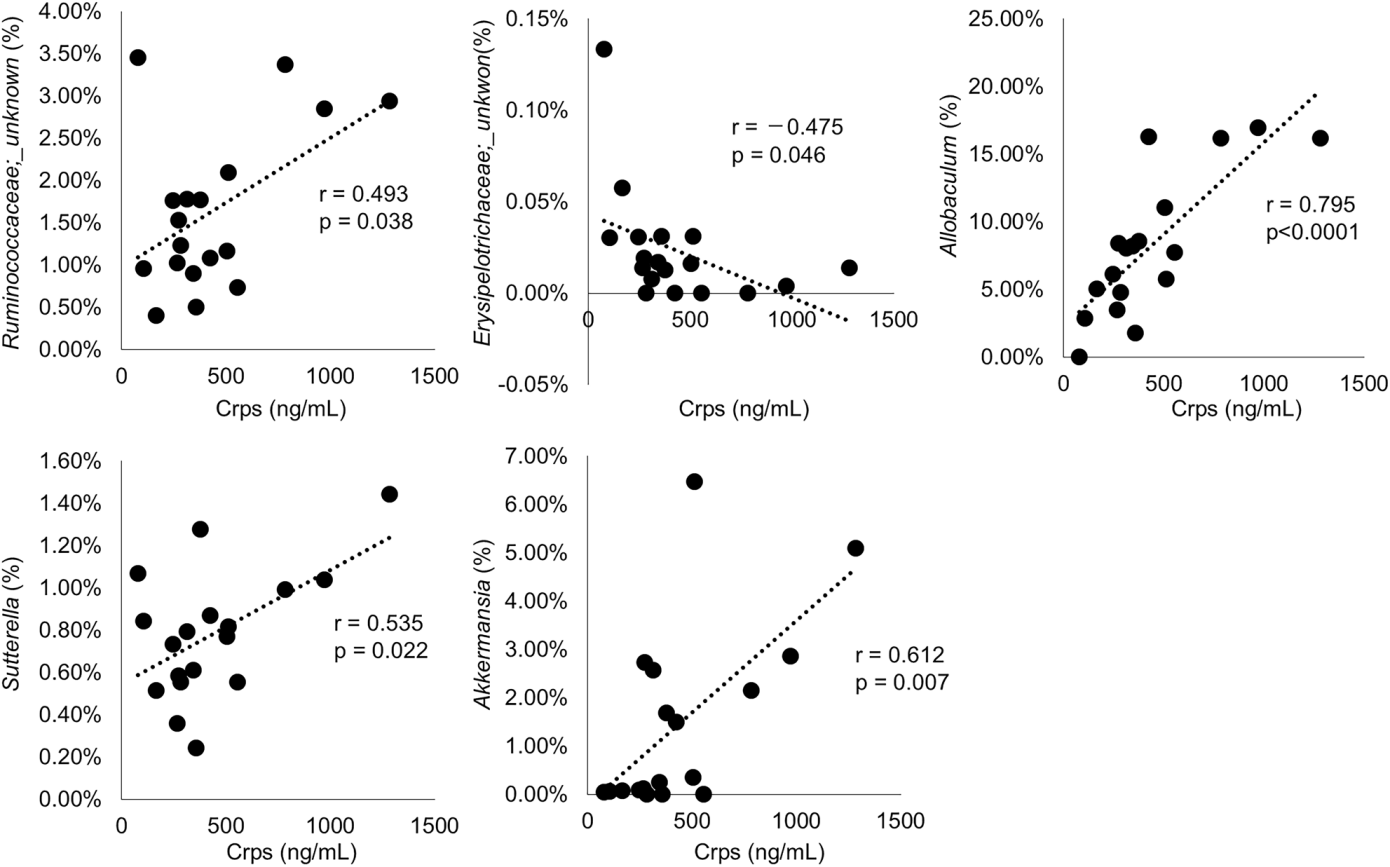
Correlation analysis between Crps and microbiota at genus level. Only pairs with significant correlations are shown in the graph. Horizontal axis shows fecal Crps concentrations after CSDS (day 32) in each mouse. All data were shown of six independent experiments in each group.

We further determined the intestinal microbiota which change significantly triggered by increase or decrease of α-defensin, by conducting correlation analyses between the amount of α-defensin in feces and the intestinal microbiota composition. *Ruminococcaceae* (r = 0.493, *P* = 0.038), *Allobaculum* (r = 0.795, *P* < 0.0001), *Sutterella* (r = 0.535, *P* = 0.022), and *Akkermansia* (r = 0.612, *P* = 0.007) showed positive correlation with α-defensin, whereas *Erysipelotrichaceae* (r = − 0.475, *P* = 0.046) showed negative correlation (Table 1, Fig. 4). Taken together, these results including the rescue experiment clarified that α-defensin reduction due to CSDS causes dysbiosis at least partially.

**Table 1:** Correlation between fecal Crps and microbiota at genus level.

Phylum	Class	Order	Family	Genus	r	*P* value
*Actinobacteria*	*Actinobacteria*	*Actinomycetales*	*Actinomycetaceae*	*Actinomyces*	0.000	1.000
			*Corynebacteriaceae*	*Corynebacterium*	0.000	1.000
			*Microbacteriaceae*	*Microbacterium*	0.000	1.000
			*Nocardiaceae*	*Rhodococcus*	0.000	1.000
		*Bifidobacteriales*	*Bifidobacteriaceae*	*Bifidobacterium*	− 0.102	0.688
	*Coriobacteriia*	*Coriobacteriales*	*Coriobacteriaceae*	*unknown*	0.096	0.704
			*Coriobacteriaceae*	*unknown*	0.070	0.784
			*Coriobacteriaceae*	*Adlercreutzia*	− 0.017	0.947
*Aquificae*	*Aquificae*	*Aquificales*	*Aquificaceae*	*Hydrogenobacter*	0.000	1.000
*Bacteroidetes*	*Bacteroidia*	*Bacteroidales*	*Bacteroidaceae*	*Bacteroides*	0.170	0.499
			*Porphyromonadaceae*	*Parabacteroides*	0.115	0.650
			*Rikenellaceae*	*unknown*	− 0.154	0.542
			*S24-7*	*unknown*	0.020	0.936
			*Paraprevotellaceae*	*Prevotella*	− 0.116	0.646
*Cyanobacteria*	*Chloroplast*	*Streptophyta*	*unknown*	*unknown*	0.000	1.000
*Deferribacteres*	*Deferribacteres*	*Deferribacterales*	*Deferribacteraceae*	*Mucispirillum*	− 0.346	0.160
*Firmicutes*	*unknown*	*unknown*	*unknown*	*unknown*	0.000	1.000
	*Bacilli*	*unknown*	*unknown*	*unknown*	0.000	1.000
		*Bacillales*	*Planococcaceae*	*Sporosarcina*	0.000	1.000
			*Staphylococcaceae*	*Jeotgalicoccus*	0.000	1.000
			*Staphylococcaceae*	*Staphylococcus*	− 0.324	0.189
		*Lactobacillales*	*Enterococcaceae*	*Enterococcus*	0.000	1.000
			*Lactobacillaceae*	*unknown*	− 0.219	0.383
			*Lactobacillaceae*	*Lactobacillus*	− 0.350	0.154
			*Leuconostocaceae*	*unknown*	0.000	1.000
			*Leuconostocaceae*	*Weissella*	0.000	1.000
			*Streptococcaceae*	*Streptococcus*	− 0.435	0.071
		*Turicibacterales*	*Turicibacteraceae*	*Turicibacter*	− 0.076	0.765
	*Clostridia*	*unknown*	*unknown*	*unknown*	− 0.237	0.343
		*Clostridiales*	*unknown*	*unknown*	− 0.251	0.316
			*unknown*	*unknown*	0.153	0.546
			*Christensenellaceae*	*unknown*	− 0.005	0.986
			*Clostridiaceae*	*Candidatus Arthromitus*	− 0.168	0.504
			*Clostridiaceae*	*Clostridium*	0.295	0.234
			*Dehalobacteriaceae*	*Dehalobacterium*	− 0.216	0.390
			*Eubacteriaceae*	*Anaerofustis*	− 0.016	0.950
			*Lachnospiraceae*	*unknown*	− 0.328	0.184
			*Lachnospiraceae*	*unknown*	− 0.283	0.256
			*Lachnospiraceae*	*Clostridium*	− 0.109	0.668
			*Lachnospiraceae*	*Coprococcus*	− 0.116	0.648
			*Lachnospiraceae*	*Dorea*	− 0.154	0.542
			*Lachnospiraceae*	*Roseburia*	− 0.203	0.420
			*Lachnospiraceae*	*Ruminococcus*	− 0.122	0.631
			*Peptococcaceae*	*unknown*	0.000	1.000
			*Peptococcaceae*	*unknown*	− 0.212	0.399
			*Peptococcaceae*	*rc4-4*	0.044	0.861
			*Ruminococcaceae*	*unknown*	− 0.308	0.213
			*Ruminococcaceae*	*unknown*	0.493	0.038*
			*Ruminococcaceae*	*Anaerotruncus*	− 0.349	0.156
			*Ruminococcaceae*	*Butyricicoccus*	− 0.238	0.341
			*Ruminococcaceae*	*Gemmiger*	− 0.343	0.164
			*Ruminococcaceae*	*Oscillospira*	− 0.351	0.153
			*Ruminococcaceae*	*Ruminococcus*	− 0.037	0.884
			*Mogibacteriaceae*	*unknown*	− 0.223	0.373
	*Erysipelotrichi*	*Erysipelotrichales*	*Erysipelotrichaceae*	*unknown*	− 0.475	0.046*
			*Erysipelotrichaceae*	*unknown*	− 0.151	0.551
			*Erysipelotrichaceae*	*Allobaculum*	0.795	< .0001**
			*Erysipelotrichaceae*	*Clostridium*	− 0.144	0.569
			*Erysipelotrichaceae*	*Coprobacillus*	− 0.250	0.317
*Proteobacteria*	*Alphaproteobacteria*	*Rhizobiales*	*Methylobacteriaceae*	*Methylobacterium*	0.000	1.000
		*Rickettsiales*	*mitochondria*	*unknown*	0.000	1.000
	*Betaproteobacteria*	*Burkholderiales*	*Alcaligenaceae*	*Sutterella*	0.535	0.022*
			*Oxalobacteraceae*	*Herbaspirillum*	0.000	1.000
	*Deltaproteobacteria*	*Desulfovibrionales*	*Desulfovibrionaceae*	*Desulfovibrio*	− 0.137	0.588
	*Gammaproteobacteria*	*Enterobacteriales*	*Enterobacteriaceae*	*unknown*	0.196	0.436
		*Pseudomonadales*	*Moraxellaceae*	*Acinetobacter*	0.000	1.000
*TM7*	*TM7-3*	*CW040*	*F16*	*unknown*	− 0.154	0.542
*Tenericutes*	*Mollicutes*	*Anaeroplasmatales*	*Anaeroplasmataceae*	*Anaeroplasma*	− 0.462	0.053
		*RF39*	*unknown*	*unknown*	− 0.100	0.695
*Verrucomicrobia*	*Verrucomicrobiae*	*Verrucomicrobiales*	*Verrucomicrobiaceae*	*Akkermansia*	0.612	0.007**
*unknown*	*unknown*	*unknown*	*unknown*	*unknown*	− 0.246	0.325

Red; *P < 0.05, **P < 0.01.

### The decrease in α-defensin due to CSDS changes fecal metabolites via dysbiosis, and the changes were recovered by Crp4 administration

Next, to determine whether intestinal metabolites were changed due to dysbiosis in the CSDS model and whether α-defensin was involved in the changes, the intestinal metabolites of CSDS group and Crp4 group were analyzed. Metabolites in feces were measured simultaneously using CE-TOFMS to identify 322 candidate compounds (Table S3). In order to clarify fecal metabolites triggered by increase or decrease of α-defensin, correlation analyses between α-defensin and the intestinal metabolites were performed. Thirty- four metabolites were positively correlated and five were negatively correlated with Crps including amino-acids and vitamins (Table 2). Sixteen amino acids or their metabolites and derivatives which showed a positive correlation include glutamic acid (r = 0.49, *P* = 0.040), lysine (r = 0.58, *P* = 0.012), and 3-amino butyric acid (r = 0.47, *P* = 0.049), alanine (r = 0.53, *P* = 0.025), allo-threonine (r = 0.57, *P* = 0.014), citrulline (r = 0.49, *P* = 0.041), isoleucine (r = 0.49, *P* = 0.038), methionine (r = 0.51, *P* = 0.030), threonine (r = 0.52, *P* = 0.028), tyrosine (r = 0.50, *P* = 0.036), β-alanine (r = 0.61, *P* = 0.007), N- acetyl glutamic acid (r = 0.56, *P* = 0.017), carnitine (r = 0.57, *P* = 0.013), isoglutamic acid (r = 0.50, *P* = 0.033), N-acetyllysine (r = 0.60, *P* = 0.008), and N5-ethylglutamine (r = 0.57, *P* = 0.013). Five vitamins or their derivatives which showed a positive correlation were nicotinic acid (r = 0.47, *P* = 0.049), pantothenic acid (r = 0.60, *P* = 0.009), pyridoxamine (r = 0.66, *P* = 0.003), pyridoxamine 5-phosphate (r = 0.50, *P* = 0.035), and thiamine phosphate (r = 0.61, *P* = 0.007). In addition, the other 13 metabolites including uracil (r = 0.51, *P* = 0.032), 1H-imidazole-4-propionic acid (r = 0.61, *P* = 0.007), and 2-hydroxypyridine (r = 0.53, *P* = 0.025), 4-methyl-2-oxovaleric acid or 3-methyl-2-oxovaleric acid (r = 0.47, *P* = 0.047), 4-methyl-5-thiazoleethanol (r = 0.63, *P* = 0.005), 5-oxo-2-tetrahydrofurancarboxylic acid (r = 0.53, *P* = 0.023), ethanolamine (r = 0.54, *P* = 0.022), fumaric acid (r = 0.56, *P* = 0.015), hexanoic acid (r = 0.48, *P* = 0.045), loperamide (r = 0.52, *P* = 0.026), malic acid (r = 0.58, *P* = 0.012), orotic acid (r = 0.53, *P* = 0.023), and succinic acid (r = 0.50, *P* = 0.035) showed a positive correlation. In contrast, five metabolites including 4-guanidinobutyric acid (r = − 0.54, *P* = 0.021), cytidine (r = − 0.48, *P* = 0.044), kynurenic acid (r = − 0.55, *P* = 0.018), N-methylproline (r = − 0.58, *P* = 0.011), and sinapic acid (r = − 0.51, *P* = 0.032) showed negative correlation with α-defensin. These results indicated that the amount of α-defensin in the intestine influences some specific intestinal metabolites.

**Table 2:** Significant correlation between fecal Crps and metabolites.

Category	Metabolites	r	*P* value
Amino acids and derivatives	Glutamic acid	0.49	0.040*
	Lysine	0.58	0.012*
	3-Aminobutyric acid	0.47	0.049*
	Alanine	0.53	0.025*
	allo-Threonine	0.57	0.014*
	Citrulline	0.49	0.041*
	Isoleucine	0.49	0.038*
	Methionine	0.51	0.030*
	Threonine	0.52	0.028*
	Tyrosine	0.50	0.036*
	β-Alanine	0.61	0.007**
	N-Acetylglutamic acid	0.56	0.017*
	Carnitine	0.57	0.013*
	Isoglutamic acid	0.50	0.033*
	N-Acetyllysine	0.60	0.008**
	N5-Ethylglutamine	0.57	0.013*
Vitamins and derivatives	Nicotinic acid	0.47	0.049*
	Pantothenic acid	0.60	0.009**
	Pyridoxamine	0.66	0.003**
	Pyridoxamine 5′-phosphate	0.50	0.035*
	Thiamine phosphate	0.61	0.007**
Others	Uracil	0.51	0.032*
	1H-Imidazole-4-propionic acid	0.61	0.007**
	2-Hydroxypyridine	0.53	0.025*
	4-Methyl-2-oxovaleric acid or 3-Methyl-2-oxovaleric acid	0.47	0.047*
	4-Methyl-5-thiazoleethanol	0.63	0.005**
	5-Oxo-2-tetrahydrofurancarboxylic acid	0.53	0.023*
	Ethanolamine	0.54	0.022*
	Fumaric acid	0.56	0.015*
	Hexanoic acid	0.48	0.045*
	Loperamide	0.52	0.026*
	Malic acid	0.58	0.012*
	Orotic acid	0.53	0.023*
	Succinic acid	0.50	0.035*
	4-Guanidinobutyric acid	-0.54	0.021*
	Cytidine	-0.48	0.044*
	Kynurenic acid	-0.55	0.018*
	N-Methylproline	-0.58	0.011*
	Sinapic acid	-0.51	0.032*

*P < 0.05, **P < 0.01.

**Figure 5:**
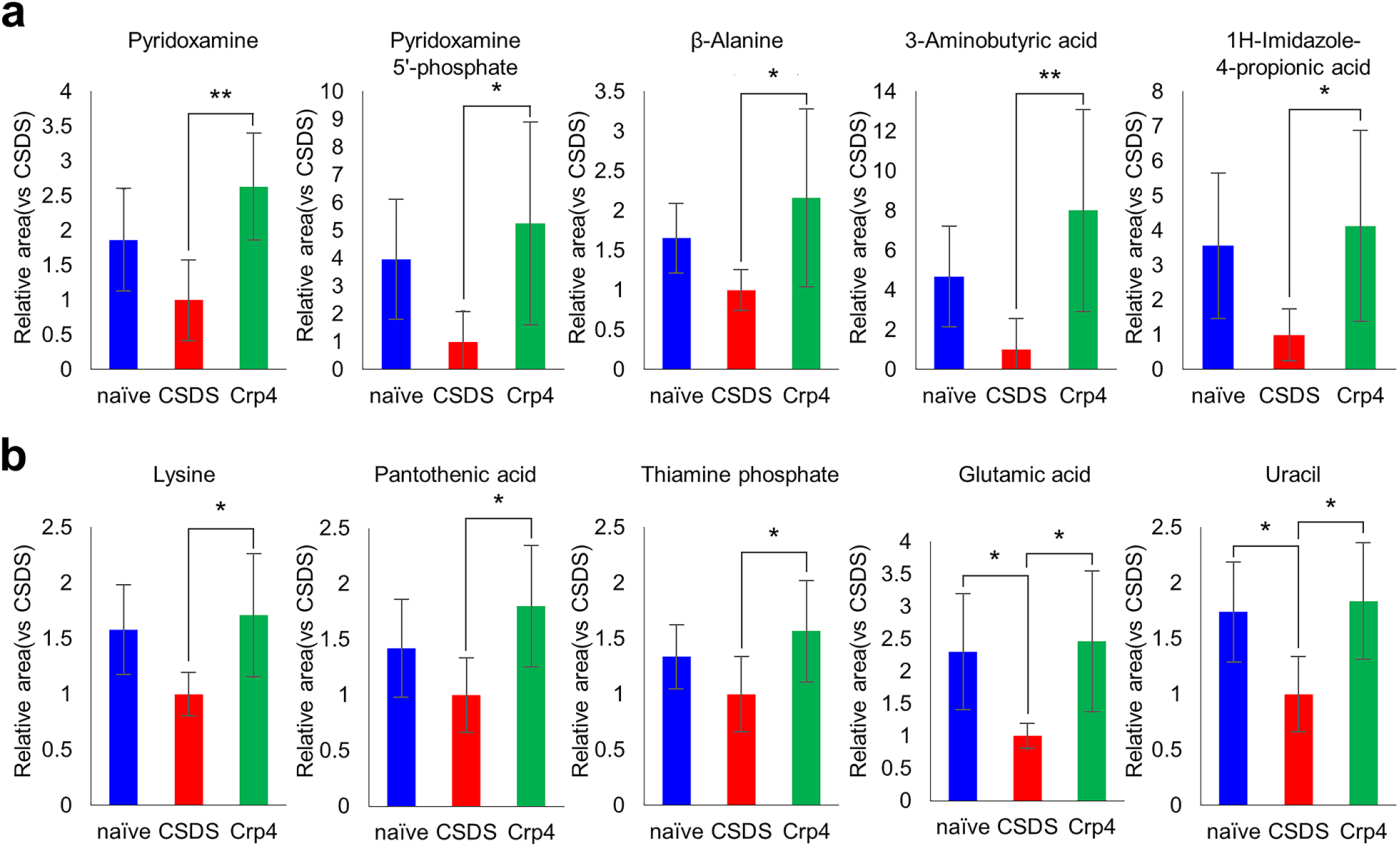
Changes in fecal metabolites correlated with Crps in each group. (**a**) Metabolites significantly correlated with Crps and microbiota (blue metabolites in Table 3). (**b**) Metabolites significantly correlated with Crps (Metabolites in Table 2 other than those shown in (**a**)). All data were shown as mean ± SE of six independent experiments in each group. Tukey's tests were used to compare the data. *P < 0.05; **P < 0.01.

We further conducted correlation analyses between the intestinal microbiota (Fig. 4) and 322 metabolites in order to know the metabolites affected by α-defensin-induced dysbiosis. Seventy-nine metabolites showed positive or negative correlation with at least one of five intestinal bacteria (Table 3). Among them, twenty-two metabolites such as pyridoxamine and β-alanine were identified as metabolites showing positive or negative correlation with α-defensin in Table 2, indicating that dysbiosis induced by α-defensin abnormalities correlates with the specific intestinal metabolites. Finally, to understand the causal relationship between α-defensin reduction and changes of the intestinal metabolites observed in the CSDS model, we analyzed differences in metabolites among each group when α-defensin was administered. Among metabolites shown in Table 2, those showed significant differences between any of the three groups were summarized, then the metabolites listed in Table 3 were shown in Fig. 5a and those not listed in Table 3 were shown in Fig. 5b.

**Table 3:** Significant correlation between microbiota which correlate with Crps and metabolites.

Metabolites	*Ruminococcaceae_unknown*		*Erysipelotrichaceae_unknown*		*Erysipelotrichaceae_Allobaculum*		*Alcaligenaceae_Sutterella*		*Verrucomicrobiaceae_Akkermansia*	
	r	*P* value	r	*P* value	r	*P* value	r	*P* value	r	*P* value value
1H-Imidazole-4-propionic acid	0.250	0.317	− 0.408	0.093	0.510	0.031*	0.097	0.701	0.133	0.598
2-Hydroxypyridine	0.555	0.017*	− 0.247	0.324	0.581	0.011*	0.278	0.264	0.204	0.417
4-Guanidinobutyric acid	− 0.296	0.233	0.175	0.488	− 0.512	0.030*	− 0.445	0.065	− 0.373	0.128
5-Oxo-2-tetrahydrofurancarboxylic acid	0.528	0.024*	− 0.238	0.342	0.573	0.013*	0.277	0.267	0.210	0.404
Alanine	0.214	0.395	− 0.375	0.125	0.496	0.036*	0.032	0.900	0.210	0.404
Carnitine	0.074	0.772	− 0.368	0.133	0.590	0.010*	− 0.024	0.925	0.254	0.310
Citrulline	0.407	0.094	− 0.151	0.549	0.485	0.042*	0.139	0.582	0.246	0.325
Ethanolamine	0.112	0.658	− 0.219	0.384	0.134	0.597	0.165	0.514	0.550	0.018*
Fumaric acid	− 0.032	0.900	− 0.374	0.126	0.469	0.050*	0.330	0.182	0.326	0.187
Isoleucine	0.206	0.412	− 0.360	0.142	0.497	0.036*	0.014	0.955	0.291	0.242
Kynurenic acid	− 0.425	0.079	0.441	0.067	− 0.531	0.023*	− 0.271	0.277	− 0.667	0.003**
Loperamide	0.560	0.016*	− 0.248	0.322	0.581	0.012*	0.277	0.265	0.202	0.422
N-Acetyllysine	0.407	0.094	− 0.263	0.292	0.541	0.020*	0.218	0.385	0.306	0.217
N-Methylproline	− 0.211	0.401	0.305	0.218	− 0.630	0.005**	− 0.330	0.181	− 0.500	0.035*
Orotic acid	0.545	0.019*	− 0.244	0.330	0.579	0.012*	0.278	0.264	0.207	0.410
Pyridoxamine	0.523	0.026*	− 0.058	0.818	0.311	0.210	0.271	0.277	0.353	0.151
Pyridoxamine 5′-phosphate	0.495	0.037*	− 0.086	0.735	0.320	0.195	0.144	0.568	0.337	0.172
Sinapic acid	− 0.282	0.257	0.331	0.180	− 0.522	0.026*	− 0.304	0.220	− 0.340	0.167
Succinic acid	0.038	0.882	− 0.463	0.053	0.508	0.032*	0.140	0.580	0.166	0.510
Tyrosine	0.221	0.378	− 0.369	0.132	0.531	0.023*	0.050	0.845	0.234	0.350
β-Alanine	0.485	0.041*	− 0.232	0.355	0.495	0.037*	0.197	0.435	0.226	0.367
3-Aminobutyric acid	0.563	0.015*	0.015	0.952	0.327	0.185	0.178	0.481	0.107	0.674
1,3-Diaminopropane	0.488	0.040*	0.296	0.233	0.172	0.495	0.533	0.023*	0.342	0.165
2′ or 5′-Deoxyadenosine	0.081	0.750	0.548	0.019*	− 0.234	0.350	0.234	0.349	0.020	0.937
2′-Deoxycytidine	0.033	0.897	0.723	0.001**	− 0.318	0.199	0.078	0.758	− 0.255	0.307
2,4-Diaminobutyric acid	0.232	0.354	0.030	0.906	− 0.070	0.784	0.023	0.928	0.557	0.016*
2,6-Diaminopimelic acid	0.566	0.014*	0.327	0.185	− 0.019	0.939	0.374	0.126	− 0.066	0.794
2-Aminoethylphosphonic acid	0.013	0.961	0.311	0.209	− 0.478	0.045*	− 0.130	0.606	− 0.061	0.810
2-Deoxyribose 1-phosphate	0.368	0.133	0.561	0.016*	− 0.070	0.782	0.205	0.414	0.001	0.998
2-Hydroxy-4-methylvaleric acid	− 0.062	0.808	− 0.575	0.013*	0.295	0.234	− 0.202	0.421	− 0.045	0.860
2-Hydroxyisobutyric acid	0.118	0.640	0.062	0.807	− 0.115	0.650	0.029	0.910	0.655	0.003**
2-Hydroxyvaleric acid	− 0.065	0.799	− 0.503	0.033*	0.271	0.277	− 0.174	0.491	− 0.066	0.796
3′-AMP	0.120	0.635	0.276	0.267	− 0.193	0.443	0.001	0.996	0.588	0.010*
3′ or 2′-CMP	0.099	0.696	0.216	0.389	− 0.204	0.417	− 0.058	0.820	0.517	0.028*
3-Phosphoglyceric acid	0.118	0.640	0.062	0.807	− 0.115	0.650	0.029	0.910	0.655	0.003**
5-Hydroxyindoleacetic acid	− 0.470	0.049*	− 0.183	0.468	− 0.179	0.478	− 0.240	0.337	− 0.261	0.295
AMP	0.068	0.790	− 0.195	0.437	0.272	0.275	0.356	0.147	0.471	0.049*
Arginine	0.131	0.605	− 0.361	0.141	0.648	0.004**	0.168	0.506	0.217	0.386
Ascorbate 2-glucoside	0.090	0.723	0.491	0.039*	− 0.429	0.076	0.009	0.972	− 0.003	0.989
Azelaic acid	− 0.288	0.247	− 0.498	0.036*	− 0.061	0.811	− 0.377	0.123	− 0.278	0.264
CMP	0.043	0.866	− 0.208	0.407	0.303	0.222	0.379	0.121	0.474	0.047*
dAMP	0.153	0.544	− 0.002	0.995	0.213	0.397	0.256	0.306	0.515	0.029*
Diphenylcarbazide	0.532	0.023*	0.429	0.076	− 0.128	0.614	0.400	0.100	0.056	0.827
Ethyl glucuronide	0.201	0.423	0.568	0.014*	− 0.517	0.028*	− 0.091	0.719	− 0.073	0.774
Fructose 6-phosphate	0.223	0.374	− 0.176	0.485	0.318	0.199	0.054	0.833	0.470	0.049*
Glutamine	0.242	0.334	− 0.287	0.248	0.613	0.007**	0.246	0.325	0.388	0.112
Glucose 6-phosphate	0.215	0.392	− 0.029	0.911	0.186	0.461	0.108	0.670	0.616	0.007**
Gly-Asp	− 0.179	0.478	− 0.535	0.022*	0.492	0.038*	0.191	0.449	0.096	0.706
Gly-Gly	− 0.161	0.525	− 0.397	0.103	0.482	0.043*	0.193	0.444	0.083	0.744
Gly-Leu	0.095	0.709	− 0.364	0.138	0.472	0.048*	0.068	0.788	0.099	0.696
Glyceric acid	− 0.519	0.027*	− 0.237	0.344	− 0.397	0.103	− 0.417	0.085	− 0.155	0.540
Homovanillic acid	− 0.032	0.901	− 0.595	0.009**	0.313	0.206	− 0.222	0.377	0.011	0.965
Hydroxyproline	− 0.146	0.564	− 0.629	0.005**	0.231	0.357	− 0.088	0.728	0.042	0.869
IsovalerylalanineN-Acetylleucine	− 0.121	0.632	− 0.655	0.003**	0.394	0.106	− 0.095	0.708	0.152	0.547
Lactic acid	− 0.282	0.256	− 0.611	0.007**	0.150	0.553	− 0.271	0.277	− 0.061	0.810
N,N-Dimethylhistidine	0.366	0.135	− 0.366	0.136	0.480	0.044*	0.364	0.137	0.549	0.018*
N-Acetylasparagine	− 0.509	0.031*	0.048	0.849	− 0.457	0.056	− 0.301	0.225	− 0.362	0.141
N-Acetylglucosamine 6-phosphate	0.474	0.047*	0.877	< .0001**	− 0.389	0.111	0.233	0.352	− 0.182	0.469
N-Acetylglucosylamine	0.028	0.912	0.572	0.013*	− 0.138	0.585	0.042	0.869	− 0.214	0.394
N-Acetylmuramic acid	0.668	0.002**	0.072	0.778	0.157	0.534	0.293	0.238	0.255	0.307
p-Hydroxymandelic acid	− 0.411	0.090	− 0.181	0.473	− 0.428	0.077	− 0.540	0.021*	− 0.152	0.547
Phenylalanine	0.138	0.586	− 0.407	0.094	0.530	0.024*	0.028	0.913	0.207	0.410
Picolinic acid	− 0.404	0.096	0.032	0.899	− 0.479	0.044*	− 0.502	0.034*	− 0.328	0.183
Pimelic acid	− 0.082	0.748	− 0.486	0.041*	0.082	0.746	− 0.247	0.324	− 0.063	0.804
Proline	− 0.372	0.129	− 0.525	0.025*	0.297	0.232	− 0.172	0.495	0.103	0.684
Putrescine	− 0.062	0.808	− 0.187	0.457	0.136	0.591	0.483	0.042*	0.244	0.330
Saccharopine	0.427	0.077	0.268	0.282	− 0.017	0.946	0.255	0.308	0.473	0.047*
Sebacic acid	− 0.233	0.353	− 0.472	0.048*	− 0.037	0.884	− 0.389	0.111	− 0.049	0.846
Sedoheptulose 7-phosphate	0.237	0.345	− 0.056	0.827	0.194	0.440	0.089	0.726	0.509	0.031*
Syringic acid	− 0.566	0.014*	− 0.294	0.237	− 0.075	0.767	− 0.618	0.006**	− 0.485	0.041*
Thiamine diphosphate	0.492	0.038*	0.009	0.971	0.217	0.387	0.150	0.554	0.375	0.125
Thymidine	0.372	0.128	0.649	0.004**	− 0.024	0.924	0.267	0.284	− 0.074	0.771
Tryptophan	− 0.110	0.665	− 0.342	0.165	0.475	0.046*	0.031	0.904	0.071	0.781
Tryptamine	0.194	0.440	0.536	0.022*	− 0.310	0.210	0.048	0.851	− 0.269	0.280
UMP	0.214	0.394	− 0.078	0.758	0.298	0.230	0.353	0.150	0.777	< .0001**
Undecanoic acid	0.476	0.046*	0.031	0.903	0.115	0.650	0.107	0.672	0.229	0.360
Urocanic acid	0.551	0.018*	0.093	0.714	0.294	0.237	0.125	0.621	0.076	0.764
Valine	0.082	0.747	− 0.395	0.105	0.471	0.048*	− 0.042	0.869	0.175	0.489
g-Glu-Val-Gly	0.070	0.782	0.736	0.001**	− 0.531	0.024*	0.038	0.881	− 0.004	0.989

Blue: metabolites correlated with Crps, red: *P < 0.05, **P < 0.01.

The metabolites significantly increased in Crp4 group relative to CSDS group were pyridoxamine (*P* = 0.002), pyridoxamine-5 phosphate (*P* = 0.027), β-alanine (*P* = 0.032), 3-aminobutyric acid, (*P* = 0.008), 1H-imidazole propionic acid (*P* = 0.044), pantothenic acid (*P* = 0.019), and thiamine-phosphoric acid (*P* = 0.044) (Fig. 5a,b). Lysine tended to decrease in CSDS group (*P* = 0.066), while significantly increased in Crp4 group (*P* = 0.023). Glutamic acid and uracil were significantly decreased in CSDS group (*P* = 0.038, *P* = 0.029), while significantly increased in Crp4 group (*P* = 0.019, *P* = 0.014) (Fig. 5b). On the other hand, no significant difference was observed between naïve group and Crp4 group for all those metabolites, indicating that the intestinal metabolites correlated with α-defensin are recovered to the same levels in naïve group by α-defensin administration. In addition, we found metabolites with significant differences in both naïve group and Crp4 group compared to CSDS group, although no correlation was observed with α-defensin (Fig. S1). Among these, three metabolites including N6-acetyllysine, penicillamine, threo-β-methylaspartic acid were significantly decreased by CSDS, and the decrease was significantly attenuated by α-defensin administration. Conversely, seven metabolites including cadaverine, glucaric acid, ferulic acid, mevalonic acid, digalacturonic acid, myo-inositol 2-phosphate, and p-aminophenol or m-aminophenol were significantly increased in CSDS group, and the increase was suppressed by α-defensin administration.

## Discussion

In this study, psychological stress reduces α-defensin secreted from Paneth cells, which induces disruption of homeostasis in the intestinal metabolites via imbalance of the intestinal microbiota, dysbiosis in CSDS model. Furthermore, the oral administration of α-defensin rescues dysbiosis and recovers homeostasis in the metabolites, suggesting that psychological stress-induced dysbiosis is associated with Paneth cell dysfunction.

We clarified that the amount of α-defensin secreted into the intestinal lumen decreases due to CSDS loading (Fig. 1f). Since the number of Paneth cells and Crp1^+^ granule area in the small intestine were decreased in CSDS group (Fig. 2a–c), the decrease in fecal Crp1 may be due to Paneth cell dysfunction. It has been known that psychological stress by mother-infant separation at birth has reduced rat Paneth cells in the small intestine via activation of corticotropin-releasing factor (CRF), and the number of Paneth cells has remained decreased even after weaning^20^. CRF activation has been also reported in CSDS model^21^. Our results that α-defensin decreased due to CSDS loading and the decrease lasted for 20 days after the end of CSDS loading are consistent with these previous findings, and importantly, abnormalities of α-defensin, the effector of innate enteric immunity, secreted by Paneth cells were first verified.

α-Defensins regulate the intestinal microbiota composition, and α-defensin deficiency diminishes diversity and affects the composition of the intestinal microbiota such as *Firmicutes* and *Bacteroidetes*, resulting in dysbiosis^14, 22, 23^. Previously, decrease in *Firmicutes* and increase in *Bacteroidetes* have been reported in patients with depression^24^. We revealed that decrease in α-defensin due to CSDS induces decrease of *Firmicutes* and increase of *Bacteroidetes* in the intestinal microbiota and further indicated a positive correlation between the amount of α-defensin and the intestinal microbiota including *Akkermansia*. It has been reported that *Akkermansia* increases by administration of α-defensin^25, 26^, and strongly correlates with stress tolerance in CSDS model^27^, suggesting α-defensin regulates the intestinal microbiota in depression. Although the status of dysbiosis varies depending on certain pathophysiology and models, because β-diversity approached to naïve group by administration of α-defensin, α-defensin may have a function to maintain homeostasis of the intestinal microbiota in response to the changes of the intestinal ecosystem.

The dysbiosis in depression has been known to be diverse between studies due to individual differences in diet, region, race, etc^28^. On the other hand, metabolic processes are relatively conserved in comparison of high variation of the intestinal microbiota between individuals^29^, indicating that individuals may have taxonomically different but functionally similar microbiota. Therefore, it is important to analyze intestinal microbial metabolites in this study. Five metabolites including pyridoxamine, pyridoxamine 5′-phosphate, β-alanine, 3-aminobutyric acid, and 1H-imidazole-4-propionic acid are significantly decreased with α-defensin reduction due to CSDS and fully recovered by α-defensin administration. Pyridoxamine, one form of vitamin B6, is important for synthesis of many neurotransmitters such as GABA, serotonin, dopamine, noradrenaline, histamine, glycine, and d-serine, and currently used as therapeutics for autism^30^. It also has been reported that vitamin B6 administration attenuates depression-like behaviors in dexamethasone-induced depression model mice^31^. In addition, β-alanine administration has been reported to improve behaviors of depression in post-traumatic stress disorder (PTSD) model mice by increasing carnosine levels in the brain and maintaining hippocampal BDNF expression, an important target of antidepressants^32, 33^. Our result that those metabolites were recovered to normal by α-defensin administration suggests that α-defensin affects brain function through microbial metabolites by maintaining homeostasis in the intestinal microbiota.

Other metabolites that decreased with α-defensin reduction due to CSDS and recovered to the same levels as naïve group by α-defensin administration include lysine, pantothenic acid, thiamine phosphate, glutamic acid, and uracil. Although these metabolites correlated with α-defensin, no direct correlation with the intestinal microbiota at genus level was observed. We think the reason why no metabolite correlates with microbiota may be because taxonomically similar bacteria often involve in the same function^29^, and further the metabolism of the intestinal microbiota can be affected by crosstalk between bacteria in addition to phenotypic changes of the bacteria themselves^34, 35^. In addition, long-term lysine deficiency has been reported to increase anxiety and psychological stress, and lysine-enriched diet interventions improve chronic anxiety^36^. Pantothenic acid has been known to increase adrenal sensitivities to adrenocorticotropic hormone (ACTH) and stimulate cortisol secretion^37^. It has been reported that people with higher erythrocyte concentration of thiamine-phosphate have lower symptoms of depression, considering thiamine-phosphate as a potential biomarker for depression^38^. Glutamic acid has been known as a major excitatory neurotransmitter that regulates higher functions such as memory and learning in the central nervous system of mammals^39^ and serves as an important material for GABA synthesis in the brain. Furthermore, it has been reported that SNPs localized in uracil-processing genes potentially regulate the onset and development of depression^40^. Taken together, the decrease of multiple intestinal metabolites which are reported to function in resolving or defending against depression, anxiety, and psychological stress links to the decrease of α-defensin, and these metabolites are recovered to the normal levels by administration of α-defensin in this study, providing the important new findings to improve understanding of depression in relation to the gut-brain axis. There are several metabolites that changed along with α-defensin reduction without correlation of the amount of α-defensin (Fig. S1). Since it has been well known that psychological stress affects many biological events in the host, it remains controversial whether biological disruptions are causes or effects. Especially, psychological stress affects the gastrointestinal function as represented in irritable bowel syndrome^41^, and the metabolism of the intestinal microbiota and the host closely interacts each other to create a complex metabolic system^34^. Thus, it is speculated that the change of these metabolites was a secondary effect of improving dysbiosis by α-defensin administration to affect the host intestinal function, and because responses to psychological stress largely vary depending on host, i.e., animal model^42^, direct correlation with α-defensin could not be observed. However, detailed underlying mechanisms of these findings remain unknown and future study is needed to further understand the gut-brain axis in depression.

In this study, we used Crp4 for administration to CSDS group because Crp4 is known to have the most potent bactericidal activities among Crps^13^ and the amount of administered Crp4 can be monitored because C57BL/6 mice do not express Crp4^43^. It has been known that Crp1 family consisted of Crp1-3 and 6, is the most abundant α-defensins in mice^44–47^. Surprisingly, Crp4 administration increased the amount of Crp1 in the CSDS mice (Fig. 3b). Given the results that Crp4 administration rescued the dysbiosis and the impaired intestinal metabolites due to CSDS loading, it is suggested that Crp4 administration improved host microenvironment for Paneth cells and resulted in the increase of Crp1 secretion. This speculation is supported by evidence on the intestinal ecosystem, including that the intestinal commensal microbiota positively affects Paneth cell development and function, i.e., Crp secretions by comparing the germ-free and the conventional mice^12, 48, 49^.

**Figure 6:**
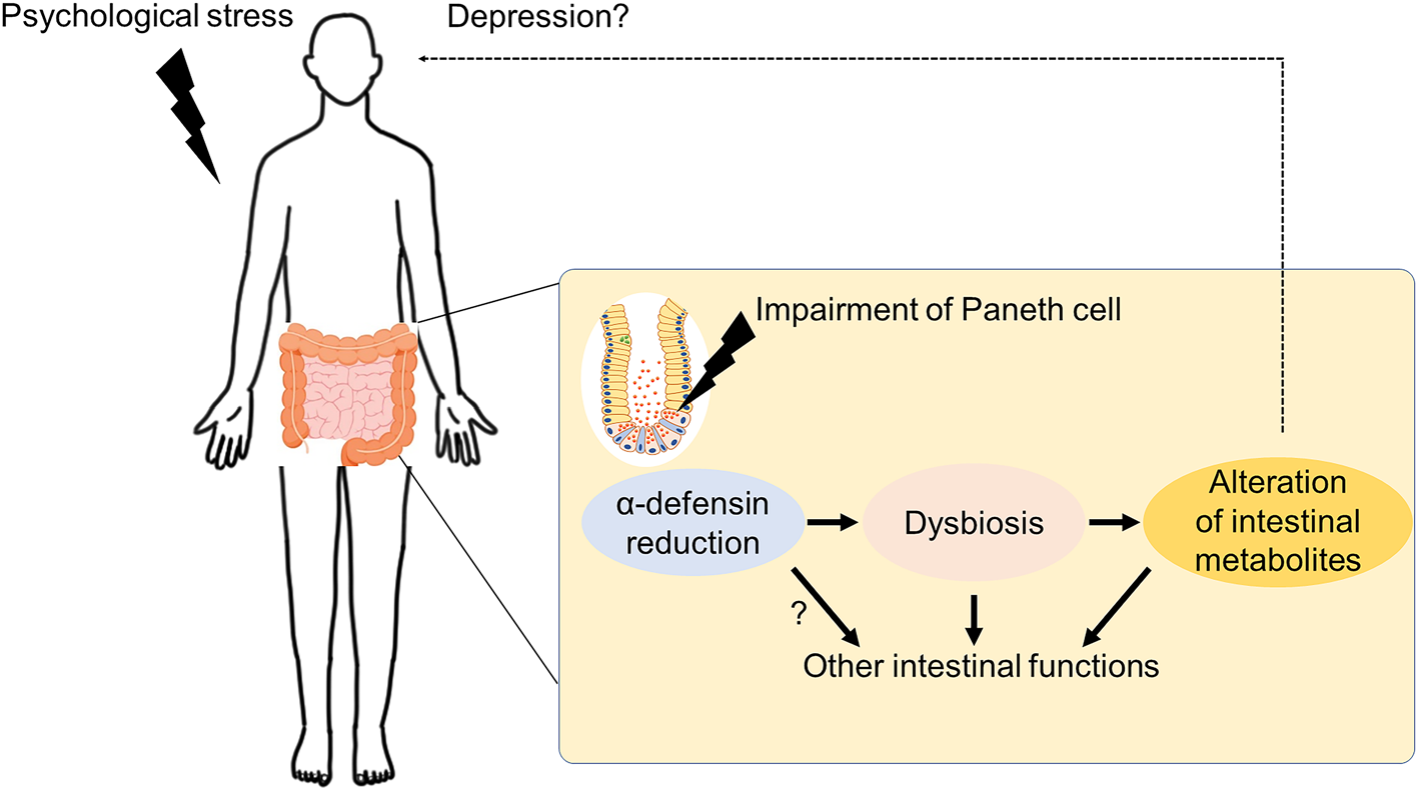
Hypothesis of a novel mechanism based on α-defensin in the CSDS model. Psychological stress reduces α-defensin produced by Paneth cells in the gut and disrupts the homeostasis of intestinal metabolite composition via dysbiosis. These are normalized by α-defensin administration. Thus, disruption of metabolite homeostasis may affect brain function and lead to the development or exacerbation of depression. However, the direct effects on the brain have not been demonstrated, and are shown as dotted lines.

This study provides a novel insight that might likely be relevant to the pathogenesis and pathophysiology of depression. Based on the results in this study, we propose a mechanism leading to depression (Fig. 6). Psychological stress immediately reduces α-defensin secretion from Paneth cells in the small intestine at early stage, resulting in dysbiosis and further disrupting homeostasis of intestinal metabolites. In the gut-brain axis, the disrupted intestinal ecosystem may affect brain through some unclarified pathways to develop or worsen depression. Dysbiosis reported in patients with depression and depression model animals largely varies probably due to individual differences relating such as diet and race^28^. This study clarified a previously unknown important link between metabolic profiles of the intestinal microbiota and upstream host-derived regulator, α-defensin, and further contribute to understanding mechanisms of depression. Although long-term observation is required to determine systemic effects including behavior in future studies to understand whole picture of the gut-brain axis in depression, the new relationship between depression and α-defensin shown in this study may contribute to development for prevention and therapeutics of depression.

## Methods

### Animal

C57BL/6 J (B6J) mice (male) and ICR mice (male) and retirement ICR (Stressor) mice (male, 5 months old and older) were purchased from Charles River Japan (Yokohama, Japan), and B6J mice and ICR mice were subjected at 7 weeks of age to experiments after acclimation and quarantine for more than one week. The bedding was PaperClean (Japan SLC Inc., Hamamatsu, Japan) and B6J mice had received ad libitum drinking water and a diet (CE-2, CLEA Japan, Tokyo, Japan). All animal experiments were approved by the Institutional Animal Care and Use Committee of the National University Corporation at Hokkaido University. All experiments were performed in accordance with Hokkaido University Regulations of Animal Experimentation. All animal experiments were also carried out in compliance with the ARRIVE guidelines.

### The experimental design of social defeat stress

CSDS was partially modified from previous reports^8^. One B6J mouse and one Stressor mouse were placed in one side of a cage divided into two compartments by a clear acrylic plate. Mice were brought into direct contact with each other. The direct contact time was counted from the time the stressor mice made contact, such as biting or covering, and the direct contact time was reduced by 5 min on the first day and by 0.5 min thereafter. In other words, the direct contact time for the 10th time was 0.5 min. Direct contact was performed at PM (13:00–17:00). After direct contact, B6J mice were moved to a neighboring compartment with stressor mice and subjected to visual and olfactory stress until the next direct contact (indirect contact). In the first experiment, five cycles of direct and indirect contact were performed, followed by two days of indirect contact, followed by the remaining five cycles each (Fig. 1a). In the second experiment, 10 cycles of direct and indirect contact were performed (Fig. 3a). Retire ICR mice were selected in order of aggression from 15 aggression tests measuring the number of 3-min bites to B6J mice performed three times a day for five days in advance.

### Behavior test

A cage (16.5 cm × 8.3 cm) was placed in a behavioral test box (50 cm × 50 cm), and the perimeter of the cage in the box was defined as the Social Interaction Zone (SI zone, cage perimeter 33.3 cm × 16.7 cm) and as both diagonal corners of the cage (Corner zone 16.6 cm × 16.6 cm). B6J mice were placed in a box with ICR mice present in the cage and allowed to explore freely for 5 min. Behavioral analysis was performed using the image analysis software HOLE BOARD (Muromachi KIKAI, Tokyo, Japan) to calculate the interaction time, corner time and total distance traveled.

### Sampling feces

Fresh feces were collected from B6J mice in cages equipped with wire mesh in the floor and stored frozen at − 80℃. Feces during 24 h were collected after cage replacement. We sampled at three time points on day 1, 9, and 14 in the first experiment (Fig. 1a) and at two time points on day 0 and 32 in the second experiment (Fig. 3a).

### Immunofluorescent analysis

Mice were euthanized by isoflurane inhalation and the small intestine was harvested at day 14 (Fig. 1a). 10% buffered formalin-fixed the small intestine from naïve and CSDS mice were embedded in paraffin and cut into 4 μm-thick sections. The sections were deparaffinized, rehydrated, and boiled in Dako REAL Target Retrieval Solution (pH 6, Agilent, Santa Clara, CA) for 20 min at 105 °C. After blocking in Block Ace (Dainippon Pharmaceutical, Osaka, Japan) containing 5% goat serum (Sigma-Aldrich, St. Louis, MO) for 30 min at room temperature, the sections were incubated with 1 μg/mL rat monoclonal anti-Crp1 (77-R63) for overnight at 4 °C. Then, the sections were incubated with 5 µg/mL Alexa Fluor 488 goat anti-rat IgG H + L (Thermo Fisher Scientific, Waltham, MA) for 1 h at room temperature. After nucleus staining by 4′, 6-diamidino-2-phenylindole (DAPI, Thermo Fisher Scientific, Waltham, MA) for 5 min, the sections were embedded to the slide grass using RapiClear 1.52 (Sunjin Lab, Hsinchu, Taiwan). Fluorescent images were observed using confocal microscopy (A1, Nikon, Tokyo, Japan). Paneth cell number per crypt and Crp1 positive area of each crypt in binary images based on fluorescent intensity were measured in 5 crypts from the small intestine by using NIS-Elements AR ver.5.11 (Nikon, Tokyo, Japan).

### α-Defensin administration

Recombinant Cryptdin-4 (mouse α-defensin) was manufactured and purified according to previous reports^50^. Samples were dissolved in ultrapure water and administered orally at 250 μg/mouse once daily from day 1 to day 32, and then equal amounts of ultrapure water were administered orally to the naïve group and the CSDS group (Fig. 3a).

### ELISA

Fecal α-defensin was measured as previously described^23, 51^. In brief, samples were air-dried, powdered using a bead beater-type homogenizer(Beads Crusher μT-12;TAITEC) . Fecal extract was collected after blending with PBS using a vortex mixer for 1 h and centrifugation at 20,000 g for 20 min, and levels of Crp1 and Crp4 were measured by sandwich ELISA. ELISA was performed on the feces of C57BL/6 J mice and the Crp1 antibodies detected Crp1-3 and 6, the Crp1 family. The Crp4 antibodies detected administered Crp4 since C57BL/6 J mice do not express Crp4 genetically.

### DNA extraction and 16S rRNA sequencing

Genomic DNA was extracted from 100 mg fecal samples using the NucleoSpin Microbial DNA Kit (MACHEREY–NAGEL, Düren, Germany) following the manufacturer’s protocol. Final DNA concentrations were determined at 260 nm using a NanoDrop 2000 spectrometer (Thermo Fischer Scientific). 16S ribosomal RNA genes were amplified by PCR from each fecal DNA sample using universal primer set of Bakt 341F (5′-cctacgggnggcwgcag) and Bakt 805R (5′-gactachvgggtatctaatcc) which covers the V3-V4 variable region^16, 52, 53^. PCR amplification was performed in 25-μl-volume reaction mixtures containing 12.5 ng of template DNA, 200 nM of each primer, and 1 × KAPA HiFi Hot Start Ready Mix (Kapa Biosystems) under the following conditions: 95 °C for 3 min, 25 cycles of 95 °C for 30 s, 55 °C for 30 s, and 72 °C for 30 s, followed by 72 °C for 5 min. PCR products were purified with AMPure XP beads (Beckman Coulter). After purification, sequencing adapters containing sample-specific 8-bp barcodes were added to the 3′- and 5′- ends by PCR using the Nextera XT Index Kit v2 Set B (Illumina) in 50 μl of reaction mixtures containing 5 μl of PCR amplicon, 5 μl of each indexing primer and 1 × KAPA HiFi Hot Start Ready Mix under the following conditions: 95 °C for 3 min, eight cycles of 95 °C for 30 s, 55 °C for 30 s, and 72 °C for 30 s, followed by 72 °C for 5 min. Each amplicon was purified, quantified using the Qubit dsDNA HS Assay Kit (Invitrogen), and then adjusted to 4 nM. Amplicons were pooled 4 μl and subjected to quantification using KAPA Library Quantification Kit LightCycler 480 qPCR Mix (Kapa Biosystems) and then diluted to 4 pM. The amplicon library was combined with 5% equimolar PhiX Control v3 (Illumina) and sequenced on a MiSeq instrument using the MiSeq 600-cycle v3 kit (Illumina).

### 16S rRNA–based taxonomic analysis

Microbiome analysis was performed using the open-source bioinformatics pipeline, QIIME2(version 2019.7). Sequences were quality filtered and denoising into features by DADA2 plugin and remaining contigs were clustered into OTUs with 99% sequence similarity against the SILVA 128 reference database. To acquire taxonomic information for each OTU, representative sequences were aligned to MAFFT and assigned to its database for classification by a naïve-bayes classifier trained on 16S rRNA gene OTUs. β-diversity (unweighted UniFrac distance) was estimated by using QIIME2 workflow. Statistical significance of β-diversity was determined by PERMANOVA test in Qiime2 pipeline.

### Measurement of fecal metabolites

Approximately 50 mg of frozen feces were plunged into 1,500 µL of 50% acetonitrile/Milli-Q water containing internal standards (H3304-1002, Human Metabolome Technologies, Inc., Tsuruoka, Japan) at 0 °C in order to inactivate enzymes. The tissue was homogenized thrice at 1,500 rpm for 120 s using a tissue homogenizer (Micro Smash MS100R, Tomy Digital Biology Co., Ltd., Tokyo, Japan) and then the homogenate was centrifuged at 2,300 × g and 4 °C for 5 min. Subsequently, 800 µL of upper aqueous layer was centrifugally filtered through a Millipore 5-kDa cutoff filter at 9,100 × g and 4 °C for 120 min to remove proteins. The filtrate was centrifugally concentrated and re-suspended in 50 µL of Milli-Q water for CE-MS analysis. Metabolome measurements were carried out through a facility service at Human Metabolome Technologies Inc., Tsuruoka, Japan. CE-TOFMS was carried out using an Agilent CE Capillary Electrophoresis System equipped with an Agilent 6210 Time of Flight mass spectrometer, Agilent 1100 isocratic HPLC pump, Agilent G1603A CE-MS adapter kit, and Agilent G1607A CE-ESI–MS sprayer kit (Agilent Technologies, Waldbronn, Germany). The systems were controlled by Agilent G2201AA ChemStation software version B.03.01 for CE (Agilent Technologies, Waldbronn, Germany). The metabolites were analyzed by using a fused silica capillary (50 μm i.d. × 80 cm total length), with commercial electrophoresis buffer (Solution ID: H3301-1001 for cation analysis and H3302-1021 for anion analysis, Human Metabolome Technologies) as the electrolyte. The sample was injected at a pressure of 50 mbar for 10 s (approximately 10 nL) in cation analysis and 25 s (approximately 25 nL) in anion analysis. The spectrometer was scanned from m/z 50 to 1000. Other conditions were as in the described previously^54^. Peaks were extracted using automatic integration software MasterHands (Keio University, Tsuruoka, Japan) in order to obtain peak information including m/z, migration time for CE-TOFMS measurement (MT) and peak area^55^. Signal peaks corresponding to adduct ions, and other product ions of known metabolites were excluded, and remaining peaks were annotated with putative metabolites and their isotopic ions from the HMT metabolite database based on their MTs and m/z values determined by TOFMS. The tolerance range for the peak annotation was configured at ± 0.5 min for MT and ± 30 ppm for m/z. In addition, peak areas were normalized against those of the internal standards and then the resultant relative area values were further normalized by sample amount.

### Statistical analysis

Statistical analysis was performed using JMP (version 14.0.0) software. Two groups were analyzed by the Mann–Whitney U test and paired t-test and three groups were analyzed by the Steel's test and Turkey's test. Correlation analysis was performed using the Pearson correlation coefficient. For all analyses, differences between groups were considered significant if P-values were < 0.05.

## Supplementary Information

Supplementary Information

## Data Availability

The datasets generated during and/or analyzed during the current study are available from the corresponding author on reasonable request.
