# Supplementary material for: Decrease of α-defensin impairs intestinal metabolite homeostasis via dysbiosis in mouse chronic social defeat stress model
Source: Sci Rep. 2021 May 10;11:9915. doi: 10.1038/s41598-021-89308-y (PMC8110768; doi:10.1038/s41598-021-89308-y)
Supplement: Supplementary file 1 — Supplementary Information [file 41598_2021_89308_MOESM1_ESM.pdf]

Supplementary Information

Decrease of α-defensin impairs intestinal metabolite homeostasis via dysbiosis in mouse chronic social defeat stress model

Kosuke Suzuki [1], Kiminori Nakamura [1,2], Yu Shimizu [2], Yuki Yokoi [2], Shuya Ohira[1], Mizu Hagiwara[1], Yi Wang [3], Yuchi Song [3], Tomoyasu Aizawa [3,4], Tokiyoshi Ayabe [1, 2, *]

[1] Innate Immunity Laboratory, Graduate School of Life Science, Hokkaido University, Sapporo, Japan. [2] Department of Cell Biological Science, Faculty of Advanced Life Science, Hokkaido University, Sapporo Japan. [3] Laboratory of Protein Science, Department of Advanced Transdisciplinary Science, Faculty of Advanced Life Science, Hokkaido University, Sapporo, Japan. [4] Global Station for Soft Matter, Global Institution for Collaborative Research and Education, Hokkaido University, Sapporo, Japan.

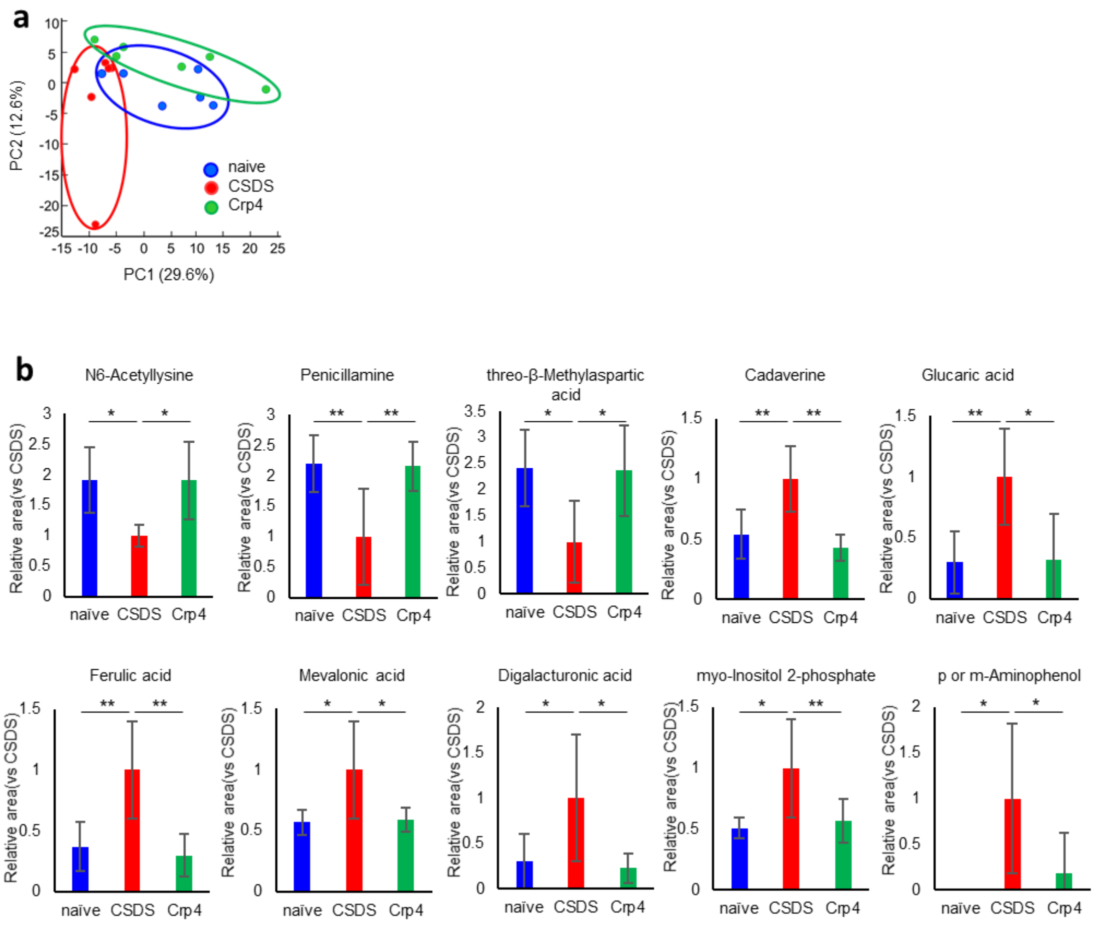

Supplementary Figure 1 Changes in fecal metabolites induced by α -defensin administration.

(a) principal component analysis of gut metabolites. (b) relative ratio of intestinal metabolites to CSDS group which are significantly different between naïve group and CSDS group, and between CSDS group and Crp4 group respectively. Data are shown as the mean ± SE. Tukey's tests were used to compare the data. *, $P < 0.05$; **, $P < 0.01$.

Supplementary Table 1 Fecal Crp1 concentration

	Day 1		Day 9		Day 14	
	Average	SD	Average	SD	Average	SD
Naïve	440.1	246.3	587.6	267.1	455.5	248.2
CSDS	231.2	216.8	146.9	15.6	198.1	90.0

(ng/g feces)

Supplementary Table 2 Abundance of microbiota (%) in each group

Phylum	Class	Order	Family	Genus	naive				CSDS				Crp4			
					before		after		before		after		before		after	
					Mean.	SD	Mean.	SD	Mean.	SD	Mean.	SD	Mean.	SD	Mean.	SD
unknown					0.02	0.02	0.01	0.01	0.03	0.02	0.01	0.01	0.06	0.08	0.02	0.02
Actinobacteria					0.78	0.35	1.36	0.42	0.82	0.32	1.75	0.91	0.70	0.20	1.77	0.70
Aquificae					0.00	0.00	0.00	0.00	0.00	0.00	0.00	0.00	0.00	0.00	0.00	0.00
Bacteroidetes					37.16	14.71	36.57	12.15	32.44	13.87	48.19	5.10	45.05	6.73	44.33	6.87
Cyanobacteria					0.00	0.00	0.00	0.00	0.00	0.00	0.00	0.00	0.00	0.00	0.00	0.00
Deferribacteres					0.25	0.15	0.05	0.03	0.48	0.51	0.04	0.03	0.19	0.13	0.03	0.02
Firmicutes					60.87	15.18	59.72	12.66	64.90	14.85	47.58	4.79	52.44	7.46	51.02	8.49
Proteobacteria					0.25	0.17	1.01	0.97	0.29	0.25	0.82	0.27	0.55	0.26	0.95	0.33
TM7					0.00	0.00	0.01	0.02	0.00	0.00	0.00	0.00	0.00	0.00	0.00	0.00
Tenericutes					0.28	0.10	0.09	0.04	0.32	0.23	0.16	0.10	0.20	0.11	0.14	0.06
Verrucomicrobia					0.39	0.54	1.18	2.60	0.72	1.06	1.44	1.16	0.81	0.87	1.74	2.04
unknown	*unknown*				0.02	0.02	0.01	0.01	0.03	0.02	0.01	0.01	0.06	0.08	0.02	0.02
Actinobacteria	*Actinobacteria*				0.00	0.01	0.18	0.43	0.00	0.00	0.47	1.08	0.00	0.00	0.00	0.00
Actinobacteria	*Coriobacteriia*				0.78	0.36	1.18	0.35	0.82	0.33	1.29	0.35	0.70	0.20	1.77	0.70
Aquificae	*Aquificae*				0.00	0.00	0.00	0.00	0.00	0.00	0.00	0.00	0.00	0.00	0.00	0.00
Bacteroidetes	*Bacteroidia*				37.16	14.71	36.57	12.15	32.44	13.87	48.19	5.10	45.05	6.73	44.33	6.87
Cyanobacteria	*Chloroplast*				0.00	0.00	0.00	0.00	0.00	0.00	0.00	0.00	0.00	0.00	0.00	0.00
Deferribacteres	*Deferribacteres*				0.25	0.15	0.05	0.03	0.48	0.51	0.04	0.03	0.19	0.13	0.03	0.02
Firmicutes	*unknown*				0.00	0.00	0.00	0.00	0.00	0.00	0.00	0.00	0.00	0.00	0.00	0.00
Firmicutes	*Bacilli*				14.42	9.57	3.04	3.60	21.61	14.05	7.38	5.79	10.63	6.18	4.17	2.04
Firmicutes	*Clostridia*				43.67	20.26	50.80	12.24	40.77	16.43	31.66	8.68	37.21	7.14	35.65	10.85
Firmicutes	*Erysipelotrichi*				2.78	3.46	5.88	3.16	2.52	2.20	8.54	4.42	4.60	2.69	11.20	6.60
Proteobacteria	*Alphaproteobacteria*				0.00	0.00	0.00	0.00	0.00	0.00	0.00	0.00	0.00	0.00	0.00	0.00
Proteobacteria	*Betaproteobacteria*				0.25	0.16	0.58	0.24	0.28	0.25	0.81	0.27	0.53	0.29	0.95	0.33
Proteobacteria	*Deltaproteobacteria*				0.00	0.00	0.43	1.05	0.00	0.00	0.00	0.00	0.00	0.00	0.00	0.00
Proteobacteria	*Gammaproteobacteria*				0.00	0.01	0.00	0.00	0.01	0.01	0.00	0.00	0.02	0.04	0.00	0.01
TM7	*TM7-3*				0.00	0.00	0.01	0.02	0.00	0.00	0.00	0.00	0.00	0.00	0.00	0.00
Tenericutes	*Mollicutes*				0.28	0.10	0.09	0.04	0.32	0.23	0.16	0.10	0.20	0.11	0.14	0.06
Verrucomicrobia	*Verrucomicrobiae*				0.39	0.54	1.18	2.60	0.72	1.06	1.44	1.16	0.81	0.87	1.74	2.04
unknown	*unknown*	*unknown*			0.02	0.02	0.01	0.01	0.03	0.02	0.01	0.01	0.06	0.08	0.02	0.02
Actinobacteria	*Actinobacteria*	*Actinomycetales*			0.00	0.01	0.00	0.00	0.00	0.00	0.00	0.00	0.00	0.00	0.00	0.00
Actinobacteria	*Actinobacteria*	*Bifidobacteriales*			0.00	0.00	0.18	0.43	0.00	0.00	0.47	1.08	0.00	0.00	0.00	0.00
Actinobacteria	*Coriobacteriia*	*Coriobacteriales*			0.78	0.36	1.18	0.35	0.82	0.33	1.29	0.35	0.70	0.20	1.77	0.70
Aquificae	*Aquificae*	*Aquificales*			0.00	0.00	0.00	0.00	0.00	0.00	0.00	0.00	0.00	0.00	0.00	0.00
Bacteroidetes	*Bacteroidia*	*Bacteroidales*			37.16	14.71	36.57	12.15	32.44	13.87	48.19	5.10	45.05	6.73	44.33	6.87
Cyanobacteria	*Chloroplast*	*Streptophyta*			0.00	0.00	0.00	0.00	0.00	0.00	0.00	0.00	0.00	0.00	0.00	0.00
Deferribacteres	*Deferribacteres*	*Deferribacterales*			0.25	0.15	0.05	0.03	0.48	0.51	0.04	0.03	0.19	0.13	0.03	0.02
Firmicutes	*unknown*	*unknown*			0.00	0.00	0.00	0.00	0.00	0.00	0.00	0.00	0.00	0.00	0.00	0.00
Firmicutes	*Bacilli*	*unknown*			0.00	0.00	0.00	0.00	0.00	0.00	0.00	0.00	0.00	0.00	0.00	0.00
Firmicutes	*Bacilli*	*Bacillales*			0.00	0.00	0.00	0.00	0.00	0.00	0.00	0.00	0.00	0.00	0.00	0.00
Firmicutes	*Bacilli*	*Lactobacillales*			14.42	9.57	3.04	3.60	21.37	14.13	7.38	5.79	10.52	6.34	3.73	2.03
Firmicutes	*Bacilli*	*Turicibacterales*			0.00	0.00	0.00	0.00	0.24	0.42	0.00	0.00	0.11	0.24	0.43	1.06
Firmicutes	*Clostridia*	*unknown*			0.00	0.00	0.00	0.00	0.00	0.00	0.00	0.01	0.00	0.00	0.00	0.00
Firmicutes	*Clostridia*	*Clostridiales*			43.67	20.26	50.80	12.23	40.76	16.42	31.66	8.68	37.21	7.14	35.65	10.85
Firmicutes	*Erysipelotrichi*	*Erysipelotrichales*			2.78	3.46	5.88	3.16	2.52	2.20	8.54	4.42	4.60	2.69	11.20	6.60
Proteobacteria	*Alphaproteobacteria*	*Rhizobiales*			0.00	0.00	0.00	0.00	0.00	0.00	0.00	0.00	0.00	0.00	0.00	0.00
Proteobacteria	*Alphaproteobacteria*	*Rickettsiales*			0.00	0.00	0.00	0.00	0.00	0.00	0.00	0.00	0.00	0.00	0.00	0.00
Proteobacteria	*Betaproteobacteria*	*Burkholderiales*			0.25	0.16	0.58	0.24	0.28	0.25	0.81	0.27	0.53	0.29	0.95	0.33
Proteobacteria	*Deltaproteobacteria*	*Desulfovibrionales*			0.00	0.00	0.43	1.05	0.00	0.00	0.00	0.00	0.00	0.00	0.00	0.00
Proteobacteria	*Gammaproteobacteria*	*Enterobacteriales*			0.00	0.01	0.00	0.00	0.01	0.01	0.00	0.00	0.02	0.04	0.00	0.01
Proteobacteria	*Gammaproteobacteria*	*Pseudomonadales*			0.00	0.00	0.00	0.00	0.00	0.00	0.00	0.00	0.00	0.00	0.00	0.00
TM7	*TM7-3*	*CW040*			0.00	0.00	0.01	0.02	0.00	0.00	0.00	0.00	0.00	0.00	0.00	0.00
Tenericutes	*Mollicutes*	*Anaeroplasmatales*			0.01	0.01	0.02	0.03	0.06	0.13	0.03	0.05	0.01	0.01	0.02	0.03
Tenericutes	*Mollicutes*	*RF39*			0.26	0.11	0.08	0.03	0.26	0.21	0.13	0.06	0.19	0.10	0.13	0.05
Verrucomicrobia	*Verrucomicrobiae*	*Verrucomicrobiales*			0.39	0.54	1.18	2.60	0.72	1.06	1.44	1.16	0.81	0.87	1.74	2.04
unknown	*unknown*	*unknown*	*unknown*		0.02	0.02	0.01	0.01	0.03	0.02	0.01	0.01	0.06	0.08	0.02	0.02
Actinobacteria	*Actinobacteria*	*Actinomycetales*	*Actinomycetaceae*		0.00	0.00	0.00	0.00	0.00	0.00	0.00	0.00	0.00	0.00	0.00	0.00
Actinobacteria	*Actinobacteria*	*Actinomycetales*	*Corynebacteriaceae*		0.00	0.00	0.00	0.00	0.00	0.00	0.00	0.00	0.00	0.00	0.00	0.00

					naive				CSDS				Crp4			
Phylum	Class	Order	Family	Genus	before		after		before		after		before		after	
					Mean.	SD	Mean.	SD	Mean.	SD	Mean.	SD	Mean.	SD	Mean.	SD
Actinobacteria	*Actinobacteria*	*Actinomycetales*	*Microbacteriaceae*		0.00	0.01	0.00	0.00	0.00	0.00	0.00	0.00	0.00	0.00	0.00	0.00
Actinobacteria	*Actinobacteria*	*Actinomycetales*	*Nocardiaceae*		0.00	0.00	0.00	0.00	0.00	0.00	0.00	0.00	0.00	0.00	0.00	0.00
Actinobacteria	*Actinobacteria*	*Bifidobacteriales*	*Bifidobacteriaceae*		0.00	0.00	0.18	0.43	0.00	0.00	0.47	1.08	0.00	0.00	0.00	0.00
Actinobacteria	*Coriobacteriia*	*Coriobacteriales*	*Coriobacteriaceae*		0.78	0.36	1.18	0.35	0.82	0.33	1.29	0.35	0.70	0.20	1.77	0.70
Aquificae	*Aquificae*	*Aquificales*	*Aquificaceae*		0.00	0.00	0.00	0.00	0.00	0.00	0.00	0.00	0.00	0.00	0.00	0.00
Bacteroidetes	*Bacteroidia*	*Bacteroidales*	*Bacteroidaceae*		1.91	0.56	0.39	0.44	1.31	0.70	0.48	0.22	2.05	0.61	0.67	0.36
Bacteroidetes	*Bacteroidia*	*Bacteroidales*	*Porphyromonadaceae*		0.92	0.26	0.63	0.25	0.74	0.39	0.71	0.40	1.16	0.53	0.64	0.16
Bacteroidetes	*Bacteroidia*	*Bacteroidales*	*Rikenellaceae*		0.00	0.00	0.19	0.45	0.00	0.00	0.00	0.00	0.00	0.00	0.00	0.00
Bacteroidetes	*Bacteroidia*	*Bacteroidales*	*S24-7*		34.33	14.62	34.30	10.79	30.38	13.18	46.34	4.92	41.84	6.34	43.01	6.77
Bacteroidetes	*Bacteroidia*	*Bacteroidales*	*[Paraprevotellaceae]*		0.00	0.00	1.07	1.22	0.00	0.00	0.66	1.23	0.00	0.00	0.00	0.00
Cyanobacteria	*Chloroplast*	*Streptophyta*	*unknown*		0.00	0.00	0.00	0.00	0.00	0.00	0.00	0.00	0.00	0.00	0.00	0.00
Deferribacteres	*Deferribacteres*	*Deferribacterales*	*Deferribacteraceae*		0.25	0.15	0.05	0.03	0.48	0.51	0.04	0.03	0.19	0.13	0.03	0.02
Firmicutes	*unknown*	*unknown*	*unknown*		0.00	0.00	0.00	0.00	0.00	0.00	0.00	0.00	0.00	0.00	0.00	0.00
Firmicutes	*Bacilli*	*unknown*	*unknown*		0.00	0.00	0.00	0.00	0.00	0.00	0.00	0.00	0.00	0.00	0.00	0.00
Firmicutes	*Bacilli*	*Bacillales*	*Planococcaceae*		0.00	0.00	0.00	0.00	0.00	0.00	0.00	0.00	0.00	0.00	0.00	0.00
Firmicutes	*Bacilli*	*Bacillales*	*Staphylococcaceae*		0.00	0.00	0.00	0.00	0.00	0.00	0.00	0.00	0.00	0.00	0.00	0.00
Firmicutes	*Bacilli*	*Lactobacillales*	*Enterococcaceae*		0.00	0.00	0.00	0.00	0.00	0.00	0.00	0.00	0.00	0.01	0.00	0.00
Firmicutes	*Bacilli*	*Lactobacillales*	*Lactobacillaceae*		14.42	9.57	3.03	3.60	21.37	14.13	7.37	5.78	10.52	6.34	3.72	2.01
Firmicutes	*Bacilli*	*Lactobacillales*	*Leuconostocaceae*		0.00	0.00	0.00	0.00	0.00	0.00	0.00	0.00	0.00	0.00	0.00	0.00
Firmicutes	*Bacilli*	*Lactobacillales*	*Streptococcaceae*		0.00	0.00	0.01	0.01	0.00	0.00	0.01	0.01	0.00	0.00	0.01	0.02
Firmicutes	*Bacilli*	*Turicibacterales*	*Turicibacteraceae*		0.00	0.00	0.00	0.00	0.24	0.42	0.00	0.00	0.11	0.24	0.43	1.06
Firmicutes	*Clostridia*	*unknown*	*unknown*		0.00	0.00	0.00	0.00	0.00	0.00	0.00	0.01	0.00	0.00	0.00	0.00
Firmicutes	*Clostridia*	*Clostridiales*	*unknown*		7.23	5.44	12.62	5.23	6.18	2.83	6.20	2.42	6.02	2.46	5.07	1.74
Firmicutes	*Clostridia*	*Clostridiales*	*unknown*		4.45	0.98	4.97	1.54	5.43	3.26	3.92	1.00	4.08	1.88	4.46	0.90
Firmicutes	*Clostridia*	*Clostridiales*	*Christensenellaceae*		0.02	0.01	0.03	0.02	0.01	0.01	0.03	0.01	0.01	0.01	0.02	0.01
Firmicutes	*Clostridia*	*Clostridiales*	*Clostridiaceae*		1.20	0.75	0.12	0.11	1.62	0.63	0.25	0.11	1.76	0.59	0.32	0.25
Firmicutes	*Clostridia*	*Clostridiales*	*Dehalobacteriaceae*		0.18	0.09	0.41	0.22	0.15	0.14	0.17	0.13	0.20	0.07	0.23	0.16
Firmicutes	*Clostridia*	*Clostridiales*	*Eubacteriaceae*		0.01	0.01	0.01	0.01	0.00	0.00	0.00	0.01	0.01	0.01	0.00	0.00
Firmicutes	*Clostridia*	*Clostridiales*	*Lachnospiraceae*		20.52	10.62	21.68	6.49	18.56	8.27	13.29	5.84	16.29	5.98	15.96	7.52
Firmicutes	*Clostridia*	*Clostridiales*	*Peptococcaceae*		0.87	0.55	0.89	0.56	1.16	0.53	1.52	0.38	0.91	0.46	1.92	0.67
Firmicutes	*Clostridia*	*Clostridiales*	*Ruminococcaceae*		9.07	4.31	9.89	3.00	7.53	3.42	6.16	2.08	7.80	1.74	7.48	1.73
Firmicutes	*Clostridia*	*Clostridiales*	*[Mogibacteriaceae]*		0.13	0.04	0.19	0.04	0.13	0.09	0.12	0.02	0.12	0.05	0.17	0.06
Firmicutes	*Erysipelotrichi*	*Erysipelotrichales*	*Erysipelotrichaceae*		2.78	3.46	5.88	3.16	2.52	2.20	8.54	4.42	4.60	2.69	11.20	6.60
Proteobacteria	*Alphaproteobacteria*	*Rhizobiales*	*Methylobacteriaceae*		0.00	0.00	0.00	0.00	0.00	0.00	0.00	0.00	0.00	0.00	0.00	0.00
Proteobacteria	*Alphaproteobacteria*	*Rickettsiales*	*mitochondria*		0.00	0.00	0.00	0.00	0.00	0.00	0.00	0.00	0.00	0.00	0.00	0.00
Proteobacteria	*Betaproteobacteria*	*Burkholderiales*	*Alcaligenaceae*		0.24	0.16	0.58	0.24	0.28	0.25	0.81	0.27	0.53	0.29	0.95	0.33
Proteobacteria	*Betaproteobacteria*	*Burkholderiales*	*Oxalobacteraceae*		0.00	0.00	0.00	0.00	0.00	0.00	0.00	0.00	0.00	0.00	0.00	0.00
Proteobacteria	*Deltaproteobacteria*	*Desulfovibrionales*	*Desulfovibrionaceae*		0.00	0.00	0.43	1.05	0.00	0.00	0.00	0.00	0.00	0.00	0.00	0.00
Proteobacteria	*Gammaproteobacteria*	*Enterobacteriales*	*Enterobacteriaceae*		0.00	0.01	0.00	0.00	0.01	0.01	0.00	0.00	0.02	0.04	0.00	0.01
Proteobacteria	*Gammaproteobacteria*	*Pseudomonadales*	*Moraxellaceae*		0.00	0.00	0.00	0.00	0.00	0.00	0.00	0.00	0.00	0.00	0.00	0.00
TM7	*TM7-3*	*CW040*	*F16*		0.00	0.00	0.01	0.02	0.00	0.00	0.00	0.00	0.00	0.00	0.00	0.00
Tenericutes	*Mollicutes*	*Anaeroplasmatales*	*Anaeroplasmataceae*		0.01	0.01	0.02	0.03	0.06	0.13	0.03	0.05	0.01	0.01	0.02	0.03
Tenericutes	*Mollicutes*	*RF39*	*unknown*		0.26	0.11	0.08	0.03	0.26	0.21	0.13	0.06	0.19	0.10	0.13	0.05
Verrucomicrobia	*Verrucomicrobiae*	*Verrucomicrobiales*	*Verrucomicrobiaceae*		0.39	0.54	1.18	2.60	0.72	1.06	1.44	1.16	0.81	0.87	1.74	2.04
unknown	*unknown*	*unknown*	*unknown*	*unknown*	0.02	0.02	0.01	0.01	0.03	0.02	0.01	0.01	0.06	0.08	0.02	0.02
Actinobacteria	*Actinobacteria*	*Actinomycetales*	*Actinomycetaceae*	*Actinomyces*	0.00	0.00	0.00	0.00	0.00	0.00	0.00	0.00	0.00	0.00	0.00	0.00
Actinobacteria	*Actinobacteria*	*Actinomycetales*	*Corynebacteriaceae*	*Corynebacterium*	0.00	0.00	0.00	0.00	0.00	0.00	0.00	0.00	0.00	0.00	0.00	0.00
Actinobacteria	*Actinobacteria*	*Actinomycetales*	*Microbacteriaceae*	*Microbacterium*	0.00	0.01	0.00	0.00	0.00	0.00	0.00	0.00	0.00	0.00	0.00	0.00
Actinobacteria	*Actinobacteria*	*Actinomycetales*	*Nocardiaceae*	*Rhodococcus*	0.00	0.00	0.00	0.00	0.00	0.00	0.00	0.00	0.00	0.00	0.00	0.00
Actinobacteria	*Actinobacteria*	*Bifidobacteriales*	*Bifidobacteriaceae*	*Bifidobacterium*	0.00	0.00	0.18	0.43	0.00	0.00	0.47	1.08	0.00	0.00	0.00	0.00
Actinobacteria	*Coriobacteriia*	*Coriobacteriales*	*Coriobacteriaceae*	*unknown*	0.07	0.02	0.04	0.04	0.05	0.08	0.04	0.06	0.07	0.05	0.04	0.03
Actinobacteria	*Coriobacteriia*	*Coriobacteriales*	*Coriobacteriaceae*	*unknown*	0.07	0.06	0.25	0.18	0.09	0.06	0.27	0.35	0.08	0.05	0.13	0.13
Actinobacteria	*Coriobacteriia*	*Coriobacteriales*	*Coriobacteriaceae*	*Adlercreutzia*	0.64	0.31	0.89	0.22	0.68	0.35	0.98	0.21	0.54	0.15	1.59	0.77
Aquificae	*Aquificae*	*Aquificales*	*Aquificaceae*	*Hydrogenobacter*	0.00	0.00	0.00	0.00	0.00	0.00	0.00	0.00	0.00	0.00	0.00	0.00
Bacteroidetes	*Bacteroidia*	*Bacteroidales*	*Bacteroidaceae*	*Bacteroides*	1.91	0.56	0.39	0.44	1.31	0.70	0.48	0.22	2.05	0.61	0.67	0.36
Bacteroidetes	*Bacteroidia*	*Bacteroidales*	*Porphyromonadaceae*	*Parabacteroides*	0.92	0.26	0.63	0.25	0.74	0.39	0.71	0.40	1.16	0.53	0.64	0.16
Bacteroidetes	*Bacteroidia*	*Bacteroidales*	*Rikenellaceae*	*unknown*	0.00	0.00	0.19	0.45	0.00	0.00	0.00	0.00	0.00	0.00	0.00	0.00
Bacteroidetes	*Bacteroidia*	*Bacteroidales*	*S24-7*	*unknown*	34.33	14.62	34.30	10.79	30.38	13.18	46.34	4.92	41.84	6.34	43.01	6.77

Phylum	Class	Order	Family	Genus	naive				CSDS				Crp4			
					before		after		before		after		before		after	
					Mean.	SD	Mean.	SD	Mean.	SD	Mean.	SD	Mean.	SD	Mean.	SD
Bacteroidetes	*Bacteroidia*	*Bacteroidales*	*[Paraprevotellaceae]*	*[Prevotella]*	0.00	0.00	1.07	1.22	0.00	0.00	0.66	1.23	0.00	0.00	0.00	0.00
Cyanobacteria	*Chloroplast*	*Streptophyta*	*unknown*	*unknown*	0.00	0.00	0.00	0.00	0.00	0.00	0.00	0.00	0.00	0.00	0.00	0.00
Deferribacteres	*Deferribacteres*	*Deferribacterales*	*Deferribacteraceae*	*Mucispirillum*	0.25	0.15	0.05	0.03	0.48	0.51	0.04	0.03	0.19	0.13	0.03	0.02
Firmicutes	*unknown*	*unknown*	*unknown*	*unknown*	0.00	0.00	0.00	0.00	0.00	0.00	0.00	0.00	0.00	0.00	0.00	0.00
Firmicutes	*Bacilli*	*unknown*	*unknown*	*unknown*	0.00	0.00	0.00	0.00	0.00	0.00	0.00	0.00	0.00	0.00	0.00	0.00
Firmicutes	*Bacilli*	*Bacillales*	*Planococcaceae*	*Sporosarcina*	0.00	0.00	0.00	0.00	0.00	0.00	0.00	0.00	0.00	0.00	0.00	0.00
Firmicutes	*Bacilli*	*Bacillales*	*Staphylococcaceae*	*Jeotgalicoccus*	0.00	0.00	0.00	0.00	0.00	0.00	0.00	0.00	0.00	0.00	0.00	0.00
Firmicutes	*Bacilli*	*Bacillales*	*Staphylococcaceae*	*Staphylococcus*	0.00	0.00	0.00	0.00	0.00	0.00	0.00	0.00	0.00	0.00	0.00	0.00
Firmicutes	*Bacilli*	*Lactobacillales*	*Enterococcaceae*	*Enterococcus*	0.00	0.00	0.00	0.00	0.00	0.00	0.00	0.00	0.00	0.01	0.00	0.00
Firmicutes	*Bacilli*	*Lactobacillales*	*Lactobacillaceae*	*unknown*	0.00	0.00	0.00	0.00	0.00	0.00	0.00	0.00	0.00	0.00	0.00	0.00
Firmicutes	*Bacilli*	*Lactobacillales*	*Lactobacillaceae*	*Lactobacillus*	14.42	9.57	3.03	3.60	21.37	14.13	7.37	5.78	10.52	6.34	3.72	2.01
Firmicutes	*Bacilli*	*Lactobacillales*	*Leuconostocaceae*	*unknown*	0.00	0.00	0.00	0.00	0.00	0.00	0.00	0.00	0.00	0.00	0.00	0.00
Firmicutes	*Bacilli*	*Lactobacillales*	*Leuconostocaceae*	*Weissella*	0.00	0.00	0.00	0.00	0.00	0.00	0.00	0.00	0.00	0.00	0.00	0.00
Firmicutes	*Bacilli*	*Lactobacillales*	*Streptococcaceae*	*Streptococcus*	0.00	0.00	0.01	0.01	0.00	0.00	0.01	0.01	0.00	0.00	0.01	0.02
Firmicutes	*Bacilli*	*Turicibacterales*	*Turicibacteraceae*	*Turicibacter*	0.00	0.00	0.00	0.00	0.24	0.42	0.00	0.00	0.11	0.24	0.43	1.06
Firmicutes	*Clostridia*	*unknown*	*unknown*	*unknown*	0.00	0.00	0.00	0.00	0.00	0.00	0.00	0.01	0.00	0.00	0.00	0.00
Firmicutes	*Clostridia*	*Clostridiales*	*unknown*	*unknown*	7.23	5.44	12.62	5.23	6.18	2.83	6.20	2.42	6.02	2.46	5.07	1.74
Firmicutes	*Clostridia*	*Clostridiales*	*unknown*	*unknown*	4.45	0.98	4.97	1.54	5.43	3.26	3.92	1.00	4.08	1.88	4.46	0.90
Firmicutes	*Clostridia*	*Clostridiales*	*Christensenellaceae*	*unknown*	0.02	0.01	0.03	0.02	0.01	0.01	0.03	0.01	0.01	0.01	0.02	0.01
Firmicutes	*Clostridia*	*Clostridiales*	*Clostridiaceae*	*andidatus Arthromitu*	1.14	0.74	0.10	0.08	1.60	0.64	0.23	0.10	1.68	0.54	0.25	0.27
Firmicutes	*Clostridia*	*Clostridiales*	*Clostridiaceae*	*Clostridium*	0.06	0.04	0.03	0.04	0.02	0.02	0.01	0.01	0.07	0.08	0.07	0.07
Firmicutes	*Clostridia*	*Clostridiales*	*Dehalobacteriaceae*	*Dehalobacterium*	0.18	0.09	0.41	0.22	0.15	0.14	0.17	0.13	0.20	0.07	0.23	0.16
Firmicutes	*Clostridia*	*Clostridiales*	*Eubacteriaceae*	*Anaerofustis*	0.01	0.01	0.01	0.01	0.00	0.00	0.00	0.01	0.01	0.01	0.00	0.00
Firmicutes	*Clostridia*	*Clostridiales*	*Lachnospiraceae*	*unknown*	16.24	8.90	13.85	4.18	15.32	7.31	9.96	3.97	12.71	6.15	12.12	5.58
Firmicutes	*Clostridia*	*Clostridiales*	*Lachnospiraceae*	*unknown*	1.31	1.04	2.32	1.08	1.03	0.69	0.99	0.73	1.13	0.66	1.20	0.93
Firmicutes	*Clostridia*	*Clostridiales*	*Lachnospiraceae*	*Clostridium*	0.27	0.52	0.16	0.21	0.11	0.15	0.06	0.04	0.08	0.07	0.09	0.09
Firmicutes	*Clostridia*	*Clostridiales*	*Lachnospiraceae*	*Coprococcus*	0.68	0.45	1.80	1.38	0.92	0.47	1.17	0.93	0.49	0.31	0.87	0.39
Firmicutes	*Clostridia*	*Clostridiales*	*Lachnospiraceae*	*Dorea*	0.01	0.01	0.03	0.04	0.01	0.02	0.02	0.03	0.02	0.02	0.03	0.03
Firmicutes	*Clostridia*	*Clostridiales*	*Lachnospiraceae*	*Roseburia*	0.12	0.11	0.23	0.34	0.12	0.14	0.17	0.16	0.10	0.15	0.16	0.19
Firmicutes	*Clostridia*	*Clostridiales*	*Lachnospiraceae*	*[Ruminococcus]*	1.89	0.99	3.28	1.26	1.05	0.74	0.91	0.53	1.76	1.07	1.50	0.93
Firmicutes	*Clostridia*	*Clostridiales*	*Peptococcaceae*	*unknown*	0.00	0.00	0.00	0.00	0.00	0.00	0.00	0.00	0.00	0.00	0.00	0.00
Firmicutes	*Clostridia*	*Clostridiales*	*Peptococcaceae*	*unknown*	0.02	0.02	0.07	0.07	0.04	0.02	0.05	0.03	0.03	0.02	0.05	0.03
Firmicutes	*Clostridia*	*Clostridiales*	*Peptococcaceae*	*rc4-4*	0.85	0.55	0.82	0.59	1.12	0.52	1.48	0.39	0.88	0.46	1.87	0.67
Firmicutes	*Clostridia*	*Clostridiales*	*Ruminococcaceae*	*unknown*	0.96	0.58	0.93	0.54	0.69	0.59	0.43	0.17	0.55	0.12	0.39	0.18
Firmicutes	*Clostridia*	*Clostridiales*	*Ruminococcaceae*	*unknown*	0.73	0.33	1.30	0.56	0.68	0.25	1.25	0.54	1.00	0.49	2.38	1.23
Firmicutes	*Clostridia*	*Clostridiales*	*Ruminococcaceae*	*Anaerotruncus*	0.15	0.09	0.29	0.12	0.16	0.12	0.12	0.08	0.11	0.07	0.11	0.08
Firmicutes	*Clostridia*	*Clostridiales*	*Ruminococcaceae*	*Butyricicoccus*	0.32	0.29	0.58	0.58	0.15	0.17	0.21	0.17	0.27	0.19	0.16	0.12
Firmicutes	*Clostridia*	*Clostridiales*	*Ruminococcaceae*	*Gemmiger*	0.48	0.60	0.05	0.04	0.14	0.15	0.04	0.02	0.12	0.03	0.04	0.02
Firmicutes	*Clostridia*	*Clostridiales*	*Ruminococcaceae*	*Oscillospira*	4.80	2.84	5.31	2.64	4.19	2.33	3.21	1.51	3.86	1.26	3.05	1.77
Firmicutes	*Clostridia*	*Clostridiales*	*Ruminococcaceae*	*Ruminococcus*	1.63	1.11	1.43	0.39	1.52	0.95	0.90	0.36	1.88	1.12	1.37	1.12
Firmicutes	*Clostridia*	*Clostridiales*	*[Mogibacteriaceae]*	*unknown*	0.13	0.04	0.19	0.04	0.13	0.09	0.12	0.02	0.12	0.05	0.17	0.06
Firmicutes	*Erysipelotrichi*	*Erysipelotrichales*	*Erysipelotrichaceae*	*unknown*	0.01	0.03	0.02	0.01	0.06	0.05	0.02	0.02	0.07	0.03	0.03	0.05
Firmicutes	*Erysipelotrichi*	*Erysipelotrichales*	*Erysipelotrichaceae*	*unknown*	1.10	0.84	0.29	0.33	0.95	0.37	0.24	0.20	1.57	0.71	0.20	0.12
Firmicutes	*Erysipelotrichi*	*Erysipelotrichales*	*Erysipelotrichaceae*	*Allobaculum*	1.23	2.48	5.51	3.15	1.25	2.06	8.21	4.55	2.45	2.05	10.89	6.76
Firmicutes	*Erysipelotrichi*	*Erysipelotrichales*	*Erysipelotrichaceae*	*Clostridium*	0.28	0.34	0.04	0.06	0.14	0.11	0.05	0.06	0.22	0.19	0.05	0.04
Firmicutes	*Erysipelotrichi*	*Erysipelotrichales*	*Erysipelotrichaceae*	*Coprobacillus*	0.15	0.16	0.02	0.03	0.12	0.06	0.01	0.02	0.29	0.30	0.03	0.04
Proteobacteria	*Alphaproteobacteria*	*Rhizobiales*	*Methylobacteriaceae*	*Methylobacterium*	0.00	0.00	0.00	0.00	0.00	0.00	0.00	0.00	0.00	0.00	0.00	0.00
Proteobacteria	*Alphaproteobacteria*	*Rickettsiales*	*mitochondria*	*unknown*	0.00	0.00	0.00	0.00	0.00	0.00	0.00	0.00	0.00	0.00	0.00	0.00
Proteobacteria	*Betaproteobacteria*	*Burkholderiales*	*Alcaligenaceae*	*Sutterella*	0.24	0.16	0.58	0.24	0.28	0.25	0.81	0.27	0.53	0.29	0.95	0.33
Proteobacteria	*Betaproteobacteria*	*Burkholderiales*	*Oxalobacteraceae*	*Herbaspirillum*	0.00	0.00	0.00	0.00	0.00	0.00	0.00	0.00	0.00	0.00	0.00	0.00
Proteobacteria	*Deltaproteobacteria*	*Desulfovibrionales*	*Desulfovibrionaceae*	*Desulfovibrio*	0.00	0.00	0.43	1.05	0.00	0.00	0.00	0.00	0.00	0.00	0.00	0.00
Proteobacteria	*Gammaproteobacteri*	*Enterobacteriales*	*Enterobacteriaceae*	*unknown*	0.00	0.01	0.00	0.00	0.01	0.01	0.00	0.00	0.02	0.04	0.00	0.01
Proteobacteria	*Gammaproteobacteri*	*Pseudomonadales*	*Moraxellaceae*	*Acinetobacter*	0.00	0.00	0.00	0.00	0.00	0.00	0.00	0.00	0.00	0.00	0.00	0.00
TM7	*TM7-3*	*CW040*	*F16*	*unknown*	0.00	0.00	0.01	0.02	0.00	0.00	0.00	0.00	0.00	0.00	0.00	0.00
Tenericutes	*Mollicutes*	*Anaeroplasmatales*	*Anaeroplasmataceae*	*Anaeroplasma*	0.01	0.01	0.02	0.03	0.06	0.13	0.03	0.05	0.01	0.01	0.02	0.03
Tenericutes	*Mollicutes*	*RF39*	*unknown*	*unknown*	0.26	0.11	0.08	0.03	0.26	0.21	0.13	0.06	0.19	0.10	0.13	0.05
Verrucomicrobia	*Verrucomicrobiae*	*Verrucomicrobiales*	*Verrucomicrobiaceae*	*Akkermansia*	0.39	0.54	1.18	2.60	0.72	1.06	1.44	1.16	0.81	0.87	1.74	2.04

Supplementary Table 3 Fecal metabolites (relative area vs internal standard)

Compound name	naive		CSDS		Crp4	
	Mean	S.D.	Mean	S.D.	Mean	S.D.
1,3-Diaminopropane	0.00062	0.00017	0.00069	0.00033	0.00095	0.00040
1-Aminocyclopropane-1-carboxylic acid Homoserinelactone	N.D.	N.D.	0.00021	N.D.	N.D.	N.D.
1-Methyl-4-imidazoleacetic acid	0.01698	0.00254	0.01502	0.00297	0.01868	0.00465
1-Methyladenosine	0.00020	0.00004	0.00028	0.00006	0.00028	0.00007
1-Methylhistidine 3-Methylhistidine	0.00177	0.00045	0.00130	0.00027	0.00192	0.00064
1*H*-Imidazole-4-propionic acid	0.01659	0.00977	0.00466	0.00346	0.01926	0.01276
2'or5'-Deoxyadenosine	0.00178	0.00076	0.00284	0.00131	0.00290	0.00182
2'-Deoxycytidine	0.00159	0.00031	0.00193	0.00063	0.00188	0.00093
2'-Deoxyguanosine	0.00088	0.00027	0.00102	0.00042	0.00132	0.00067
2,4-Diaminobutyric acid	0.00105	0.00122	0.00055	N.D.	0.00072	0.00035
2,5-Dihydroxybenzoic acid	0.00026	0.00007	0.00041	0.00025	0.00023	0.00003
2,5-Pyrroledione	0.00047	0.00008	0.00072	0.00030	0.00071	0.00001
2,6-Diaminopimelic acid	0.00348	0.00036	0.00366	0.00104	0.00477	0.00085
2-(Creatinine-3-yl)propionic acid	0.00250	0.00039	0.00275	0.00066	0.00277	0.00040
2-Amino-2-(hydroxymethyl)-1,3-propanediol	0.00050	0.00012	0.00061	0.00015	0.00055	0.00010
2-Amino-2-methyl-1,3-propanediol	0.00042	0.00003	0.00037	0.00004	0.00047	0.00008
2-Aminoethylphosphonic acid	0.00030	0.00014	0.00023	0.00010	0.00022	0.00006
2-Aminoisobutyric acid 2-Aminobutyric acid	0.01132	0.00416	0.00758	0.00301	0.01013	0.00314
2-Deoxyglucose 6-phosphate	0.00029	0.00006	0.00017	N.D.	0.00027	0.00011
2-Deoxyribose 1-phosphate	0.00201	0.00059	0.00163	0.00087	0.00295	0.00117
2-Hydroxy-4-methylvaleric acid	0.00738	0.00340	0.00454	0.00159	0.00524	0.00337
2-Hydroxybutyric acid	0.00075	0.00035	0.00027	0.00007	0.00087	0.00072
2-Hydroxyisobutyric acid	0.00013	N.D.	N.D.	N.D.	N.D.	N.D.
2-Hydroxypyridine	N.D.	N.D.	N.D.	N.D.	0.00011	0.00001
2-Hydroxyvaleric acid	0.00307	0.00148	0.00154	0.00046	0.00216	0.00139
2-Isopropylmalic acid	0.00033	0.00011	0.00039	0.00011	0.00020	0.00009
2-Methylserine	0.00044	0.00022	0.00035	0.00003	0.00051	0.00008
2-Oxoglutaric acid	0.01308	0.00695	0.00565	0.00175	0.01401	0.00670
2-Oxoisovaleric acid	0.00415	0.00184	0.00237	0.00056	0.00352	0.00138
3'-AMP	0.00039	0.00035	0.00018	0.00005	0.00016	0.00003
3'-CMP 2'-CMP	0.00050	0.00058	0.00024	0.00014	0.00029	0.00010
3,4-Dihydroxyphenylglycol	0.00031	0.00005	0.00031	0.00002	0.00031	0.00007
3-(4-Hydroxyphenyl)propionic acid	0.01299	0.00511	0.02230	0.01493	0.00681	0.00218
3-Amino-2-piperidone	0.00039	0.00008	0.00039	0.00011	0.00038	0.00008
3-Aminobutyric acid	0.00078	0.00016	0.00041	0.00006	0.00110	0.00070
3-Aminoisobutyric acid	0.00099	0.00024	0.00112	0.00039	0.00112	0.00037
3-Aminopropane-1,2-diol	0.00029	N.D.	0.00048	0.00021	0.00034	0.00008
3-Dehydroshikimic acid	0.00019	0.00006	0.00018	0.00005	0.00014	0.00003
3-Hydroxy-3-methylglutaric acid	0.00042	0.00007	0.00047	0.00016	0.00030	0.00004
3-Hydroxybutyric acid	0.00145	0.00098	0.00048	0.00017	0.00079	0.00074
3-Hydroxypropionic acid	0.00132	0.00038	N.D.	N.D.	0.00159	0.00025
3-Methoxytyrosine	0.00029	0.00007	N.D.	N.D.	0.00026	N.D.
3-Methylguanine	0.00059	0.00009	0.00050	0.00015	0.00060	0.00012
3-Phenylpropionic acid	0.00952	0.00353	0.00610	0.00127	0.00872	0.00247
3-Phosphoglyceric acid	0.00021	N.D.	N.D.	N.D.	N.D.	N.D.
4-(β-Acetylaminoethyl)imidazole	0.00041	0.00034	0.00045	0.00025	0.00084	0.00089
4-Acetamidobutanoic acid	0.00020	0.00008	0.00014	N.D.	0.00028	0.00008
4-Guanidinobutyric acid	0.00037	0.00008	0.00038	0.00009	0.00023	N.D.
4-Methyl-2-oxovaleric acid 3-Methyl-2-oxovaleric acid	0.01813	0.00679	0.01155	0.00206	0.01713	0.00636
4-Methyl-5-thiazoleethanol	0.00018	0.00006	0.00017	0.00006	0.00022	0.00009
4-Methylthio-2-oxobutyric acid	0.00054	0.00016	0.00022	0.00006	0.00042	0.00015
4-Pyridoxic acid	0.00257	0.00024	0.00238	0.00043	0.00246	0.00043

Compound name	naive		CSDS		Crp4	
	Mean	S.D.	Mean	S.D.	Mean	S.D.
Compound name	Control		Stress		Stress+Crp4	
	Mean	S.D.	Mean	S.D.	Mean	S.D.
5-Aminovaleric acid	0.121962	0.058019	0.065527	0.060502	0.051636	0.031686
5-Hydroxyindoleacetic acid	0.00025	9.38E-05	0.000318	8.07E-05	0.000262	0.000125
5-Hydroxylysine	0.001598	0.000181	0.001918	0.00112	0.001549	0.000404
5-Methoxyindoleacetic acid	0.00013	4.52E-05	0.00014	3.44E-05	0.000143	2.86E-05
5-Methyl-2'-deoxycytidine	0.000412	7.13E-05	0.00042	8.14E-05	0.000425	1E-04
5-Methylcytosine	0.000148	N.D.	0.000154	N.D.	N.D.	N.D.
5-Oxo-2-tetrahydrofurancarboxylic acid	N.D.	N.D.	N.D.	N.D.	0.000208	6E-05
5-Oxoproline	0.002184	0.00109	0.001006	0.000206	0.002091	0.001122
6-Aminohexanoic acid	0.003918	0.001162	0.002886	0.000233	0.003941	0.00108
6-Hydroxyhexanoic acid	0.000778	0.000153	0.000553	0.000223	0.000562	0.000328
6-Hydroxynicotinic acid	0.000321	0.000151	0.000178	8.19E-05	0.000457	0.000308
7-Methylguanine	0.00148	0.00047	0.000798	0.000201	0.001433	0.000533
8-Hydroxyoctanoic acid	0.000133	3.21E-05	0.000146	3.12E-05	0.000129	2.61E-05
Acetoacetic acid	0.000345	1.52E-06	9.82E-05	N.D.	0.000292	0.000195
Adenine	0.00791	0.00439	0.015402	0.007931	0.00817	0.003894
Adenosine	0.00131	0.000632	0.002799	0.001287	0.00234	0.001388
Adipic acid	N.D.	N.D.	N.D.	N.D.	0.000249	N.D.
Ala	0.398698	0.108875	0.340303	0.065558	0.434838	0.140068
allo-Threonine	0.000706	0.00017	0.000584	0.00013	0.000743	0.000244
Alloisoleucine	0.003497	0.002071	0.002386	0.000602	0.003021	0.001628
AMP	0.00012	2.5E-05	0.000124	1.33E-06	0.000146	N.D.
Anserine_divalent	0.000491	5.44E-05	0.000442	9.16E-05	0.000554	5.61E-05
Arg	0.011764	0.002765	0.0125	0.008677	0.013648	0.004047
Arg-Glu	0.000777	0.000206	0.000594	0.000155	0.000904	0.000279
Argininosuccinic acid	N.D.	N.D.	0.000232	5.88E-05	0.000145	3.57E-05
Ascorbate 2-glucoside	0.00062	0.000145	0.000613	0.000169	0.000726	0.000116
Ascorbic acid	0.000159	6.78E-05	0.000181	4.15E-06	0.000123	1.96E-05
Asn	0.000503	N.D.	0.000932	0.000372	0.000657	0.000135
Asp	0.164454	0.098352	0.1024	0.04329	0.140979	0.072573
Azelaic acid	0.002671	0.000855	0.002128	0.000592	0.002133	0.001003
Azetidine 2-carboxylic acid	N.D.	N.D.	0.000548	N.D.	N.D.	N.D.
Betaine	0.005847	0.000978	0.006558	0.002688	0.005662	0.00153
Betaine aldehyde_+H_2O	0.000376	0.000118	0.000417	0.000205	0.000345	4.92E-05
Betonicine	N.D.	N.D.	0.000339	0.000114	N.D.	N.D.
Cadaverine	0.00036	0.000137	0.000665	0.000179	0.000285	7.35E-05
cAMP	0.000173	4.49E-05	0.000158	3.9E-05	0.000161	3.53E-05
Carboxymethyllysine	0.001095	0.000223	0.000819	0.000145	0.001113	0.000325
Carnitine	0.001184	0.000325	0.001181	0.000276	0.001322	0.000346
Cholic acid	0.160209	0.091828	0.168993	0.071684	0.184877	0.035942
Choline	0.026136	0.004774	0.031314	0.005141	0.020295	0.006615
Cimetidine	0.000657	0.000143	0.000768	0.000269	0.00072	5.37E-05
cIMP	0.00013	4.95E-05	0.000135	3.12E-05	0.000144	1.96E-05
cis-4-Hydroxyproline	0.000713	0.000258	0.00046	7.5E-05	0.000595	0.000241
Citraconic acid	0.00037	9.66E-05	0.000168	2.47E-05	0.000359	0.000164
Citramalic acid	0.000984	0.000298	0.000464	4.94E-05	0.000786	8.94E-05
Citric acid	0.007782	0.004426	0.00706	0.002208	0.004829	0.001351
Citrulline	0.025209	0.008405	0.020051	0.008897	0.031702	0.011937
CMP	0.000136	1.34E-05	0.000156	6.32E-05	0.000224	N.D.
CMP-*N*-acetylneuraminate	N.D.	N.D.	7.51E-05	N.D.	N.D.	N.D.
Creatine	0.000279	7.02E-05	0.004302	0.007819	0.00024	7.51E-05
Creatinine	0.000428	0.000133	0.001208	0.001492	0.000556	0.000118
Crotonic acid	0.00019	1.2E-05	0.000224	N.D.	N.D.	N.D.
Cyclohexanecarboxylic acid	0.000127	1.87E-05	0.000137	5.33E-05	N.D.	N.D.

Compound name	naive		CSDS		Crp4	
	Mean	S.D.	Mean	S.D.	Mean	S.D.
Cysteic acid	0.00182	0.000681	0.001128	0.000221	0.001973	0.000756
Cystine	0.00015	2.21E-05	0.000244	N.D.	0.000196	N.D.
Cytidine	0.003416	0.000496	0.003758	0.001215	0.003442	0.000882
Cytosine	0.001805	0.002059	0.00252	0.002674	0.000963	0.000344
Daminozide<br>Ala-Ala	0.011289	0.004895	0.004629	0.001741	0.010773	0.005418
dAMP	0.000244	9.42E-05	0.000203	5.32E-05	0.00017	5.8E-05
dCMP	0.000348	0.000207	0.00037	0.000291	0.00037	0.00023
Diethanolamine	0.000777	0.00052	0.001787	0.001552	0.000949	0.00065
Digalacturonic acid	0.000368	0.000197	0.000801	0.00056	0.000219	0.000107
Dihydroxyacetone phosphate	0.000738	0.000204	0.000457	0.000266	0.000623	0.000215
Diphenylcarbazide	0.004342	0.000415	0.004317	0.001126	0.005229	0.000534
Dodecanedioic acid	0.002253	0.000684	0.001541	0.000596	0.001643	0.000852
dTMP	0.000287	0.000128	0.000326	0.000218	0.000375	0.000136
Dyphylline	0.004477	0.002493	0.00652	0.003419	0.005896	0.003583
Ectoine	0.000519	0.000384	0.000575	0.000208	N.D.	N.D.
Erythrose 4-phosphate	0.000124	N.D.	N.D.	N.D.	0.000132	N.D.
Ethanolamine	0.019544	0.003553	0.01593	0.00397	0.017658	0.005333
Ethyl glucuronide	0.001471	0.000178	0.001391	0.000354	0.00163	0.000212
Ferulic acid	0.002634	0.001427	0.007124	0.002859	0.002101	0.001247
FMN	0.000167	4.67E-05	N.D.	N.D.	0.000213	3.84E-05
Formiminoglutamic acid	0.001621	0.000734	0.00089	0.000233	0.001729	0.000601
Fructose 6-phosphate	0.000412	0.000268	0.000182	6.82E-05	0.000373	0.00038
Fumaric acid	0.004364	0.001782	0.004348	0.001627	0.004619	0.003404
GABA	0.005402	0.001075	0.005799	0.001368	0.008411	0.007119
Galactosamine<br>Glucosamine	0.001852	0.001723	0.001163	0.00045	0.002623	0.002076
Gln	0.031105	0.012118	0.026783	0.016563	0.033027	0.009169
Glu	0.680785	0.265032	0.295855	0.057193	0.728424	0.321606
Glu-Glu	0.000816	0.00024	0.000737	0.000222	0.000737	0.000165
Glucaric acid	0.000135	4.05E-05	0.000302	0.000119	0.000191	7.37E-05
Gluconic acid	0.001858	0.000981	0.001834	0.000593	0.001121	0.000461
Gluconolactone	0.001318	0.000455	0.001401	7.88E-05	0.00127	0.000101
Glucosamine 6-phosphate	0.000135	N.D.	N.D.	N.D.	N.D.	N.D.
Glucose 1-phosphate	0.000681	0.00034	0.00021	6.24E-05	0.000627	0.000492
Glucose 6-phosphate	0.000929	0.001268	0.000464	0.000463	0.000541	0.00072
Glucuronic acid-1<br>Galacturonic acid-1	0.002598	0.000928	0.002654	0.000629	0.00186	0.000256
Glucuronic acid-2<br>Galacturonic acid-2	0.012901	0.005743	0.01471	0.0046	0.007941	0.001004
Glutaric acid	0.001023	0.000268	0.000943	0.000332	0.000966	0.000306
Gly	0.086458	0.026444	0.061047	0.019617	0.07631	0.024031
Gly-Asp	0.001115	0.000216	0.001203	0.000267	0.001114	0.000366
Gly-Gly	0.001176	0.00018	0.001329	0.000525	0.00108	0.000204
Gly-Leu	0.003396	0.000838	0.003511	0.001856	0.003546	0.001179
Glyceric acid	0.011923	0.003287	0.011125	0.002635	0.006561	0.001177
Glycerol	0.173539	0.030253	0.194803	0.049306	0.190731	0.035331
Glycerol 2-phosphate	0.000116	1.19E-05	N.D.	N.D.	N.D.	N.D.
Glycerol 3-phosphate	0.000877	0.000204	0.001227	0.000371	0.000835	0.000345
Glycolic acid	0.003042	0.00052	0.001861	0.000741	0.001793	0.000233
GMP	N.D.	N.D.	9.56E-05	N.D.	N.D.	N.D.
Guanine	0.002296	0.000696	0.00368	0.001307	0.003577	0.001996
Guanosine	0.000762	0.00044	0.001154	0.000594	0.001506	0.000776
Heptanoic acid	0.000234	1.55E-05	0.000166	3.04E-05	N.D.	N.D.
Hexanoic acid	0.000701	0.000277	0.000418	0.000103	0.001059	0.000782
His	0.019968	0.003811	0.017665	0.007798	0.015841	0.002864
His-Glu	0.000209	5.46E-05	0.000194	8.18E-05	0.00024	5.57E-05

Compound name	naive		CSDS		Crp4	
	Mean	S.D.	Mean	S.D.	Mean	S.D.
Histamine	0.000193	N.D.	0.000243	N.D.	0.000528	0.000205
Histidinol	N.D.	N.D.	N.D.	N.D.	7.8E-05	N.D.
Homocarnosine	0.000135	3.47E-05	0.000147	2.19E-05	0.000117	2.48E-06
Homocitrulline	0.000822	0.000128	0.000722	0.000199	0.000762	0.00022
Homocysteic acid	0.000105	1.18E-05	0.00011	1.74E-05	0.000116	2.34E-05
Homoserine	0.006423	0.002163	0.003212	0.000698	0.006893	0.003136
Homovanillic acid	0.00078	0.000273	0.000405	6.02E-05	0.000785	0.000315
Hydroxyindole	0.001887	0.000222	0.001856	0.000297	0.00215	0.000481
Hydroxyproline	0.010616	0.004134	0.008793	0.002994	0.007968	0.00395
Hypotaurine	0.000435	0.000151	0.000408	3.02E-05	0.000454	7.16E-05
Hypoxanthine	0.159686	0.059208	0.076001	0.030919	0.147222	0.048871
Ile	0.152056	0.033504	0.110904	0.04063	0.139863	0.044727
Imidazole-4-acetic acid	N.D.	N.D.	0.00033	N.D.	0.000336	0.000308
Imidazole-4-methanol	0.000481	0.000213	0.000527	0.000125	0.000479	0.000256
Imidazolelactic acid	0.000275	0.000113	0.000471	0.00017	0.000362	0.000176
Indole-3-acetic acid	0.000274	7.34E-05	0.000315	8.43E-05	0.000231	8.23E-05
Inosine	0.008574	0.003988	0.005917	0.005153	0.016578	0.014507
Isethionic acid	0.00122	0.002072	0.000381	0.00022	0.000369	0.000171
Isoamylamine	N.D.	N.D.	N.D.	N.D.	0.005593	N.D.
Isobutyric acid<br>Butyric acid	0.105586	0.049927	0.064962	0.044534	0.113579	0.090151
Isocitric acid	0.000433	N.D.	N.D.	N.D.	N.D.	N.D.
Isoglutamic acid	0.016566	0.010423	0.004554	0.001918	0.019063	0.014994
Isopropanolamine	0.001486	0.000193	0.002205	0.000765	0.001486	0.000477
Isovaleric acid<br>Valeric acid	0.029833	0.010352	0.018039	0.002843	0.025816	0.011199
Isovalerylalanine<br>*N*-Acetylleucine	0.000214	5.13E-05	0.000159	1.3E-05	0.00019	4.64E-05
Kojic acid	0.001075	0.000215	0.001008	0.000262	0.001022	0.000124
Kynurenic acid	0.000109	2.1E-05	0.000114	3.39E-05	0.000141	9.53E-06
Lactic acid	0.195511	0.083869	0.153629	0.059531	0.111622	0.067228
Lauric acid	0.000195	9.84E-06	0.000152	2.99E-05	0.000151	N.D.
Leu	0.213415	0.040676	0.156594	0.061918	0.198728	0.064978
Loperamide	N.D.	N.D.	N.D.	N.D.	0.000326	4.54E-06
Lys	0.209441	0.053193	0.132497	0.025826	0.226391	0.073281
Malic acid	0.063788	0.031826	0.055595	0.020562	0.066251	0.034661
Melatonin	0.001397	0.000107	0.001377	0.000279	0.001533	0.000107
Met	0.072054	0.018464	0.046387	0.013598	0.070259	0.02717
Methionine sulfoxide	0.006163	0.001484	0.004171	0.000474	0.006236	0.001709
Mevalolactone	0.002477	N.D.	0.002103	0.00012	N.D.	N.D.
Mevalonic acid	0.000279	5E-05	0.00049	0.000195	0.000289	4.81E-05
Mucic acid	0.000218	7.87E-06	0.000282	3.01E-05	0.00019	N.D.
myo-Inositol 1-phosphate<br>*myo*-Inositol 3-phosphate	0.000556	0.000234	0.000319	0.000126	0.000413	9.3E-05
myo-Inositol 2-phosphate	0.000354	5.89E-05	0.000699	0.000281	0.000395	0.000128
N,*N*-Dimethylglycine	0.002287	0.001859	0.002308	0.00207	0.002134	0.00101
N,*N*-Dimethylhistidine	0.00028	1.47E-05	0.000297	0.000127	0.00033	6.54E-05
N-Acetyl-β-alanine	0.00064	0.00011	0.000603	0.000191	0.000422	0.000184
N-Acetylalanine	0.000257	7.75E-05	0.000201	5.51E-05	0.000204	5.37E-05
N-Acetylasparagine	0.00015	2.71E-05	0.000165	8.62E-05	0.000164	7.57E-05
N-Acetylaspartic acid	0.000435	9.18E-05	0.000488	0.000184	0.00044	0.00023
N-Acetylgalactosamine<br>*N*-Acetylmannosamine<br>*N*-Acetylglucosamine	0.036627	0.009249	0.042755	0.014919	0.04456	0.018249
N-Acetylglucosamine 1-phosphate	0.000114	1.32E-07	0.000175	N.D.	N.D.	N.D.
N-Acetylglucosamine 6-phosphate	N.D.	N.D.	N.D.	N.D.	6.41E-05	N.D.
N-Acetylglucosylamine	N.D.	N.D.	0.000376	6.77E-05	0.000433	0.000173

Compound name	naive		CSDS		Crp4	
	Mean	S.D.	Mean	S.D.	Mean	S.D.
N-Acetylglutamic acid	0.003557	0.001297	0.001981	0.000789	0.00356	0.001163
N-Acetylglutamine	0.000436	4.73E-05	0.000433	6.07E-05	0.000432	8.77E-05
N-Acetylglycine	0.000189	0.000151	0.000364	0.000315	0.000125	N.D.
N-Acetylhistidine	0.001543	0.000777	0.001162	0.00037	0.000861	0.000167
N-Acetyllysine	0.007959	0.001934	0.006389	0.000992	0.01063	0.004778
N-Acetylmethionine	0.00044	0.000117	0.000333	9.34E-05	0.000368	0.000112
N-Acetylmuramic acid	0.00355	0.001157	0.003086	0.001082	0.005008	0.001853
N-Acetylneuraminic acid	0.019562	0.004925	0.022195	0.004558	0.017563	0.00176
N-Acetylornithine	0.004638	0.001137	0.003273	0.00052	0.004652	0.001858
N-Acetylputrescine	0.002605	0.001086	0.003179	0.001718	0.001485	0.000906
N-Acetylserine	0.001775	0.001107	0.003345	0.002088	0.001072	0.000339
N-Acetyltryptophan	0.000442	1.52E-05	0.000432	7.46E-05	0.000482	5.03E-05
N-Carbamoylaspartic acid	0.000174	6.5E-05	0.000237	N.D.	0.000214	3.46E-06
N-Formylmethionine	0.000202	2.44E-05	0.000207	2.55E-05	0.000185	2.54E-05
N-Methylalanine	0.005981	0.003004	0.002038	0.000966	0.006244	0.005136
N-Methylglutamic acid	0.000708	0.000502	0.000321	6.93E-05	0.000594	0.00038
N-Methylnorsalsolinol	0.000501	0.000103	0.000456	8.75E-05	0.000512	0.000167
N-Methylproline	0.005333	0.003299	0.003691	0.002838	0.002802	0.003034
N^2-Acetylaminoadipic acid	0.000545	0.000251	0.000263	2.77E-05	0.000466	0.000184
N^2-Phenylacetylglutamine	0.000686	0.00012	0.000646	0.000144	0.000769	8.72E-05
N^2-Succinylornithine	0.000985	0.000546	0.001245	0.00043	0.001576	0.001286
N^5-Ethylglutamine	0.0023	0.001104	0.001233	0.000478	0.002178	0.001002
N^6,*N*^{6},*N*^{6}-Trimethyllysine	0.001008	0.000396	0.00058	0.000111	0.000821	0.000357
N^6-Acetyllysine	0.001799	0.000506	0.00094	0.000167	0.001796	0.0006
N^6-Methyladenine	0.000416	9.4E-05	0.000369	9.02E-05	0.000431	0.000143
N^6-Methyllysine	0.000935	0.000319	0.00066	0.000151	0.000911	0.000346
N^8-Acetylspermidine	0.001291	0.000648	0.000777	0.000353	0.001257	0.000528
Nicotinic acid	0.074068	0.024796	0.042703	0.013337	0.075163	0.027114
N_ω-Methylarginine	0.00013	N.D.	N.D.	N.D.	N.D.	N.D.
O-Acetylhomoserine<br>2-Aminoadipic acid	0.000886	0.000189	0.000987	0.000259	0.000807	6.9E-05
O-Acetylserine	0.000596	0.000265	0.00066	0.000506	0.000465	0.000187
o-Coumaric acid<br>*p*-Coumaric acid	0.000217	4.94E-05	0.000284	0.000105	0.000146	2.35E-05
o-Hydroxybenzoic acid	0.000249	9.28E-05	0.000309	0.000216	0.000192	4.32E-05
Octanoic acid	0.000209	N.D.	N.D.	N.D.	0.000338	N.D.
Ornithine	0.082143	0.019759	0.057638	0.021198	0.079585	0.034459
Orotic acid	N.D.	N.D.	N.D.	N.D.	0.001144	0.000187
Oxypurinol	N.D.	N.D.	N.D.	N.D.	0.003444	N.D.
p-Aminobenzoic acid	0.001149	0.00035	0.000647	0.000196	0.001146	0.000596
p-Aminophenol<br>*m*-Aminophenol	N.D.	N.D.	0.000345	7.56E-05	0.000249	N.D.
p-Hydroxybenzoic acid	0.000256	5.99E-05	0.000528	0.000448	N.D.	N.D.
p-Hydroxymandelic acid	0.000368	7.24E-05	0.000325	7.4E-05	0.000305	5.49E-05
p-Hydroxyphenylacetic acid	0.008522	0.003999	0.006748	0.003459	0.005116	0.002353
p-Hydroxyphenylpyruvic acid	0.000859	0.000188	0.00058	0.000128	0.000651	0.00025
p-Toluic acid<br>*m*-Toluic acid<br>*o*-Toluic acid	0.001382	0.00034	0.001051	0.000532	0.001224	0.000274
Pantothenic acid	0.010582	0.003282	0.00746	0.0025	0.01344	0.004075
Penicillamine	0.000829	0.000175	0.000564	6.46E-05	0.000812	0.000152
Phe	0.108583	0.021767	0.082683	0.0395	0.1009	0.035863
Phenylpyruvic acid	0.001124	0.000489	0.000575	0.000141	0.000928	0.000392
Phosphorylcholine	0.000613	0.000446	0.0009	0.000266	N.D.	N.D.
Phthalic acid	0.000243	4.23E-05	0.000258	5.51E-05	0.000252	3.44E-05
Picolinic acid	0.000372	7.68E-05	0.000267	N.D.	N.D.	N.D.
Pimelic acid	0.000367	6.01E-05	0.000332	8.91E-05	0.000347	0.000106

Compound name	naive		CSDS		Crp4	
	Mean	S.D.	Mean	S.D.	Mean	S.D.
Pipecolic acid	0.00492	0.001541	0.004539	0.001558	0.003969	0.001398
Piperidine	0.000912	0.000359	0.000783	0.00019	0.000733	0.000267
Pro	0.134677	0.018226	0.121447	0.023889	0.117042	0.027328
Propionic acid	0.071227	0.020905	0.04873	0.016245	0.045379	0.009561
Prostaglandin E_2	0.003168	0.000407	0.003026	0.000603	0.003444	0.000164
Purine	0.000418	8.24E-05	N.D.	N.D.	0.000413	0.000102
Putrescine	0.005162	0.001861	0.008505	0.004716	0.005416	0.002695
Pyridoxal	0.002003	0.000145	0.001602	0.000336	0.001731	0.000195
Pyridoxamine	0.00161	0.000636	0.000862	0.0005	0.002271	0.00066
Pyridoxamine 5'-phosphate	0.000531	0.00029	0.000268	5.39E-06	0.000703	0.000488
Pyridoxine	0.001876	0.001199	0.002139	0.000934	0.001117	0.00041
Pyrophosphate	0.012274	0.001499	0.009672	0.001973	0.012245	0.002482
Pyruvic acid	0.003029	0.000861	0.003779	0.001547	0.003196	0.000814
Quinic acid	0.001182	0.000118	0.001181	0.000234	0.001265	0.000158
Quinolinic acid	0.00041	0.000136	0.000361	9.51E-05	0.000498	0.000175
Riboflavin	0.0009	8.24E-05	0.000738	0.000165	0.001052	0.000265
Ribose 5-phosphate	0.00079	0.000561	0.000471	0.000378	0.000744	0.000647
Ribulose 5-phosphate	0.002644	0.001373	0.002505	0.001651	0.004632	0.003063
S-Adenosylmethionine	0.000573	N.D.	0.000372	0.000233	0.001918	0.001415
S-Sulfocysteine	0.000234	6.19E-05	0.000376	0.000172	0.000239	6.57E-05
Saccharopine	0.000847	0.000251	0.000654	0.000115	0.000927	0.000258
Sarcosine	0.007153	0.002514	0.008578	0.007167	0.005303	0.001168
SDMA	0.000655	0.000128	0.000529	0.000148	0.000549	0.000143
Sebacic acid	0.001223	0.000244	0.001	0.000288	0.000941	0.000407
Sedoheptulose 7-phosphate	0.001796	0.001594	0.000545	0.00038	0.001159	0.001578
Ser	0.059905	0.007109	0.053825	0.016166	0.053348	0.011878
Ser-Glu	0.001668	0.000287	0.001351	0.000336	0.001853	0.000891
Serotonin	0.000323	9.14E-05	0.000401	0.000122	0.000437	8.22E-05
Sinapic acid	0.0008	0.000113	0.000905	0.000181	0.000816	9.01E-05
Spermidine	0.020068	0.009368	0.011338	0.007048	0.024574	0.010878
Stachydrine	0.00055	0.000166	0.000711	0.000326	0.00044	0.00014
Suberic acid	0.001265	0.000177	0.001201	0.000328	0.00121	0.000344
Succinic acid	0.110001	0.064798	0.062137	0.036094	0.0913	0.043954
Syringic acid	0.000217	6.23E-05	0.000276	5.48E-05	0.00021	5.86E-05
Taurine	0.04152	0.050556	0.047825	0.049037	0.037053	0.025805
Taurocholic acid	0.001032	0.00088	0.002503	0.002473	0.0007	0.000218
Terephthalic acid	0.000707	0.00011	0.000755	3.64E-05	0.000734	4.62E-05
Thiamine	0.001734	0.000342	0.000996	0.000423	0.001912	0.000822
Thiamine diphosphate	0.000194	0.000103	0.000108	N.D.	0.000237	9.81E-05
Thiamine phosphate	0.000604	0.00013	0.000451	0.000153	0.000707	0.000205
Thr	0.089341	0.020491	0.061793	0.015146	0.088605	0.026827
Thr-Asp	0.000938	0.000118	0.000806	0.000207	0.001001	0.000433
threo-β-Methylaspartic acid	0.000735	0.000225	0.000457	4.33E-05	0.00072	0.000265
Threonic acid	N.D.	N.D.	0.00063	7.93E-05	0.000687	0.000268
Thymidine	0.002798	0.000324	0.002798	0.000668	0.003666	0.001029
Thymine	0.014199	0.003962	0.014153	0.00665	0.017247	0.006135
trans-Glutaconic acid	0.005726	0.002964	0.001807	0.00075	0.004943	0.003579
Trehalose 6-phosphate	0.000196	8.02E-06	N.D.	N.D.	0.000147	N.D.
Trigonelline	N.D.	N.D.	0.000771	N.D.	N.D.	N.D.
Trimethylamine	0.035522	0.011909	0.022105	0.006717	0.033877	0.008928
Trimethylamine *N*-oxide	0.000442	7.48E-05	0.000274	4.33E-05	0.000282	5.28E-05
Tropic acid<br>3-Phenyllactic acid<br>3-(2-Hydroxyphenyl)propionic acid<br>*m*-Ethoxybenzoic acid<br>*p*-Methoxyphenylacetic acid<br>Atrolactic acid	0.00156	0.000797	0.000908	0.000171	0.001056	0.000625

Compound name	naive		CSDS		Crp4	
	Mean	S.D.	Mean	S.D.	Mean	S.D.
Trp	0.01009	0.00071	0.012529	0.011027	0.0091	0.00296
Tryptamine	N.D.	N.D.	N.D.	N.D.	0.000183	1.09E-05
Tyr	0.058287	0.01331	0.041661	0.020115	0.057451	0.023289
Tyr-Glu	0.000351	2.94E-05	0.000317	6.85E-05	0.000382	0.000101
Tyramine	0.000356	9.39E-05	0.000385	0.000104	0.000591	0.000251
UMP	0.000213	N.D.	0.000126	N.D.	0.000153	N.D.
Undecanoic acid	0.000138	1.6E-05	0.000125	3.13E-05	0.000162	2.35E-05
Uracil	0.034032	0.008771	0.019551	0.006578	0.035936	0.010285
Uridine	0.007276	0.002367	0.004355	0.001334	0.008383	0.00421
Urocanic acid	0.00431	0.000463	0.003566	0.000476	0.00565	0.002149
Val	0.174883	0.038409	0.127777	0.05845	0.155071	0.047241
Xanthine	0.067344	0.021117	0.035805	0.01071	0.061896	0.020195
Xanthosine	0.000593	0.000248	0.000292	0.000185	0.000947	0.001211
Xanthurenic acid	N.D.	N.D.	0.000129	N.D.	0.000225	N.D.
β-Ala	0.012548	0.0033	0.00758	0.00194	0.016364	0.008494
β-Ala-Lys	0.000344	0.00019	0.000287	0.000115	0.000676	0.000673
β-Tyr	0.000232	N.D.	0.000185	6.56E-05	0.000189	N.D.
γ-Butyrobetaine	0.015485	0.006745	0.008516	0.001836	0.015222	0.008677
γ-Glu-2-aminobutyric acid	0.000791	0.00033	0.000578	0.000141	0.000843	0.000264
γ-Glu-Val-Gly	0.001324	0.000331	0.001407	0.000273	0.001539	0.000538

N.D.: not detected.

For peaks that were not identified as a single metabolite, multiple candidates are described.
